# Notch mediated lateral inhibition is shaped by morphological differences to reinforce bias toward signal-sending or receiving roles

**DOI:** 10.1016/j.cub.2026.07.041

**Published:** 2026-08-12

**Authors:** Prachi Richa, Charalambos Roussos, Chengxi Zhu, Martin O. Lenz, Shahar Kasirer, David Sprinzak, Sarah Bray

**Affiliations:** 1 Department of Physiology Development and Neuroscience, University of Cambridge, Downing Street, Cambridge, CB2 3DY, UK; 2 Cambridge Advanced Imaging Centre, University of Cambridge, Downing Street, Cambridge, CB2 3DY, UK; 3 School of Physics and Astronomy, Tel Aviv University, Tel Aviv 69978, Israel; 4 School of Neurobiology, Biochemistry and Biophysics, Tel Aviv University, Tel Aviv 69978, Israel

**Keywords:** Notch, Lateral inhibition, live-transcription, cell-mechanics, mathematical modelling

## Abstract

During neurogenesis, neuroblasts are selected from proneural-competent cells through lateral inhibition, a process controlled by the evolutionarily conserved Notch signalling pathway. By tracking transcription from Notch-target genes and cell morphologies in real time, we discovered that the presumptive neuroblast never initiates target-gene transcription. This implies a pre-existing bias directs Notch signalling. The bias correlates with a heterogeneity in apical cell areas which is further reinforced during neuroblast selection. Additionally, the length and duration of neuroblast-neighbour cell contacts predict the likelihood of transcription. Using mathematical modelling we show that lateral inhibition seeded with subtle morphological differences can bias cells toward signal-sending or receiving roles before transcriptional feedback occurs. Notch activation further alters apical cell area, reinforcing the initial bias. We propose that signalling and cell mechanics work together to ensure the robust selection of a single neural precursor.

## Introduction

The selection of neural precursor cells from proneural-competent cells is regulated by the evolutionarily conserved Notch signalling pathway through lateral inhibition^1^. One feature of models explaining this process is an intercellular transcriptional feedback regulatory loop^1–3^ in which proneural proteins upregulate Notch ligands, while Notch activation induces HES family repressors (*Enhancer of split*, [*E(spl)*] genes, in *Drosophila*), which suppress proneural proteins and ligand expression. This feedback can amplify small initial differences, resulting in an all-or-none switch where one cell retains proneural activity and becomes the neural stem cell, whereas its neighbours are prevented from doing so^1^. While there is evidence to support many aspects of this feedback mechanism, several observations challenge our understanding of how it operates in the real time of many developmental fate decisions^4,5^. Notably it is unclear whether all cells have equal potential, and a stochastic event tips the balance in favour of one cell retaining neural determinants, or whether there is a pre-existing bias on which lateral inhibition operates.

It is also evident that, while transcriptional feedback is important, regulation of Delta and Notch can occur on multiple levels. For example, selection of neural precursors occurs normally under conditions where uniform Notch or Delta expression is provided, arguing there are other modes of regulation^6–8^. Mechanisms that regulate Delta activity more directly, including the E3 ubiquitin ligase Neuralized, likely participate in the molecular switch^9–11^. In addition, cis-inhibition, inhibitory interactions between ligands and receptors present on the same cell surface^12–14^, modulates Notch activity levels in many contexts including adult sensory organs, where it sets a baseline of activity for the selection of a single precursor^14^. Finally, properties of contacts between cells can affect levels and durations of cell-to-cell signalling^15,16^.

Signalling is normally initiated when transmembrane ligands on one cell bind Notch receptors on a neighbour^17^. The ligand exerts a mechanical pulling force, likely generated by its endocytosis, that triggers a series of proteolytic cleavages leading to release of the Notch intracellular domain, NICD^18^. Released NICD enters the nucleus and forms activation complexes that drive transcription of target genes including HES family genes^19^. The mechanical nature means that signalling is influenced by physical properties like tissue stiffness, junctional tensions, intracellular contractility^20 21^. As neuroblast (NB) selection during *Drosophila* embryogenesis unfolds within a dynamically remodelling neuroepithelium and the NB undergoes apical constriction and Myosin-II–dependent ingression^22,23^, mechanical regulation could contribute to Notch-mediated lateral inhibition during this process. However, whether there is an interplay between morphogenetic changes and spatiotemporal signalling within proneural clusters remains unknown.

To investigate Notch signalling dynamics during lateral inhibition, we combined real-time imaging of Notch target-gene transcription^24,25^ with quantitative morphodynamic analysis, laser ablation, and genetic perturbations. Strikingly, we found a pre-existing bias whereby the presumptive NB never initiates Notch target-gene transcription, correlating with pre-existing differences in apical cell area that become reinforced during NB selection. Incorporating these dynamic area differences into a mathematical model reproduced the observed transcriptional patterns. Together, our findings suggest that lateral inhibition is initiated from a landscape with subtle heterogeneities that bias cells toward signal-sending or signal-receiving states before transcriptional feedback, with signalling and cell mechanics acting together to ensure robust selection of a single neural precursor.

## Results

### Notch-responsive transcription reveals an early bias within proneural clusters

**Figure 1:**
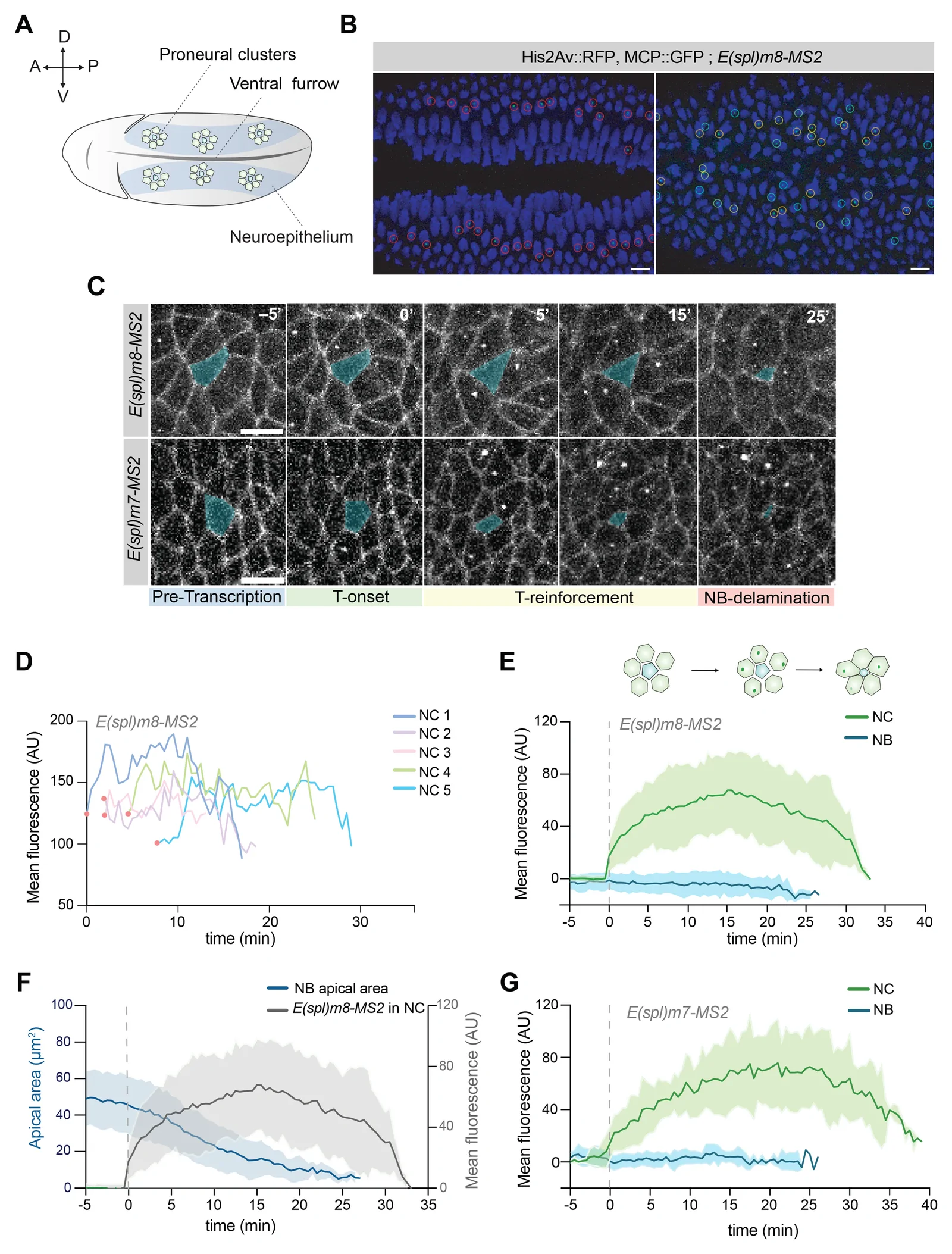
Tracking Notch-responsive transcription in neighbour-clusters reveals an early transcriptional bias **(A)** Schematic illustrating proneural clusters within ventral neuroectoderm (blue shading) flanking the ventral furrow in stage 7–8 *Drosophila* embryo. Dorsal (D), ventral (V), Anterior (A) and posterior (P) axis are indicated. **(B)** Images from in vivo movie, *E(spl)m8–MS2* transcription puncta (green) within nuclei (blue, H2Av::RFP) in mesectoderm (left, red circles) prior to ventral closure, and in ventral neuroectoderm (right, yellow and blue circles). Scale bars in this and subsequent images =10µm. **(C)** Time-lapse images from neighbour-cluster transcribing *E(spl)m8–MS2* or *E(spl)m7–MS2*, Spider::GFP marks cell membranes. Presumptive NB (cyan), identified by back-tracking from delamination (25’ right side). Transcription is aligned by time when *E(spl)m8/m7-MS2/*MCP puncta first detected (T-Onset 0’*)*. Images at times relative to onset (0’) as indicated. **(D)** *E(spl)m8-MS2*/MCP intensity profiles from individual cells within a representative cluster. **(E)** Mean fluorescence intensity of *E(spl)m8-MS2/MCP* in NCs (green) and presumptive NBs (blue) aligned by T-onset within a cluster (time 0). N=30 clusters, 84 NCs. **(F)** Comparison between *E(spl)m8-MS2* transcription per cluster (grey, replicated from E) and NB apical area (blue). Average NB-delamination duration, 18.43±4.90 mins. **(G)** Mean fluorescence intensity of *E(spl)m7-MS2*/MCP aligned to T-onset (time 0) as in E. N=11 clusters, 34 NCs. See also Figure S1, Video S1, Video S2 and Table S1.

Each NB originates from a proneural cluster (Figure 1A, S1A)^26,27^. To investigate whether all cells in a cluster initially signal equivalently to one another, before one acquires a higher signalling potential, or whether a bias pre-patterns signalling, we set out to track Notch-dependent transcriptional dynamics in real time within proneural clusters. We introduced MS2 loops into an endogenous Notch target gene by CRISPR engineering, producing *E(spl)m8–MS2* flies^25^. When combined with fluorescent MS2 binding protein, MCP::GFP, discrete transcription foci give a direct measure of the RNA molecules being transcribed (Figure 1B)^28^. We focussed on the first S1 wave of neurogenesis which initiates just after mesoderm invagination is completed (stage 7-8 embryos; Figure 1B; S1A)^26^ and identified delaminating NBs using a membrane marker (Spider::GFP) to track the decrease in their apical area. The associated neurogenic clusters could then be mapped by back-tracking (Figure 1C). We refer to these as “neighbour-clusters”.

To characterize transcription dynamics at single-cell resolution, we performed 30–40-minute time-lapse imaging and extracted intensity of transcription foci in all tracked cells from identified neighbour-clusters (Figure 1C, Video S1). First, comparing times when foci first appeared, revealed that transcription initiated asynchronously within and between clusters (Figure 1C,D; S1B) and that within cluster lag times were ~5–7 minutes. We then aligned traces based on onset of detectable transcription within each cluster (time 0, T-onset). *E(spl)m8–MS2* transcription lasted for 25–30 minutes per cluster (25.90 ± 4.90 min, mean ± SD), with an average duration per nucleus of circa 15 minutes (Figure 1E; S1C). During this period, the NB apical area decreased until it fully delaminated (Figure 1C,F). The final stage of NB ingression was accompanied by an asynchronous loss in cluster transcription, which persisted in some cells for 5–6 minutes longer (Figure 1F).

To investigate whether all cells within a neighbour-cluster, including the NB, initially activate transcription, they were backtracked to a stage several minutes prior to appearance of the first transcription foci. We then assessed transcriptional activity in the presumptive NB and neighbouring cells (NCs) of each cluster (Figure 1C,E; Video S2). Based on previous analysis, we estimate that foci with 3 RNAs are above the detection limit^24^ yet no foci were ever visible in NBs either before or after transcription initiated in NCs (Figure 1E). These results argue that there is a bias within the proneural cluster that precedes onset of *E(spl)m8* transcription and shapes Notch signalling within clusters.

We next asked whether a second Notch-responsive gene, *E(spl)m7*, exhibited the same transcriptional dynamics, using a similar *E(spl)m7–MS2* line^24^. When aligned by T-onset, *E(spl)m7–MS2* produced comparable transcription profile intensities and durations to *E(spl)m8–MS2* (32.23 ± 5.60 mins; Figure 1G, S1C; Video S1). Importantly, *E(spl)m7–MS2* was only transcriptionally active in NCs and was silent in the presumptive NB throughout the entire signalling window.

Notch-responsive transcription is thus highly dynamic over the timescale of the NB fate decision. Absence of detectable *E(spl)m8–MS2* and *E(spl)m7–MS2* transcription in the presumptive NB argues that there is a bias in signalling from the outset. As both had similar properties we focused on *E(spl)m8–MS2* for subsequent experiments.

### Presumptive NB is the signalling source for cells in direct contact

Two models could explain the pattern of transcription observed. One that the presumptive NB is the signalling source, activating Notch in NCs in direct contact. The other that NCs signal to each other while the NB is blocked from responding.

To test experimentally which cells provide the signal, we performed targeted laser ablations during the period when transcription first initiates (0–5 minutes), removing either the NB or an NC. Proneural clusters were identified using a reporter containing 3kb proximal *achaete* regulatory sequences upstream of multimerised Halo coding sequences (*ac-Halo*) and were first detectable prior to midline closure (stage 6/7; Figure S1A). *ac-Halo* was combined with *E(spl)m8–MS2* and, at the stage when transcription foci first appeared, ablations within an *ac-Halo* cluster were focused on the NB or an adjacent NC based on cell positions. The cluster was then tracked to monitor the consequences.

**Figure 2:**
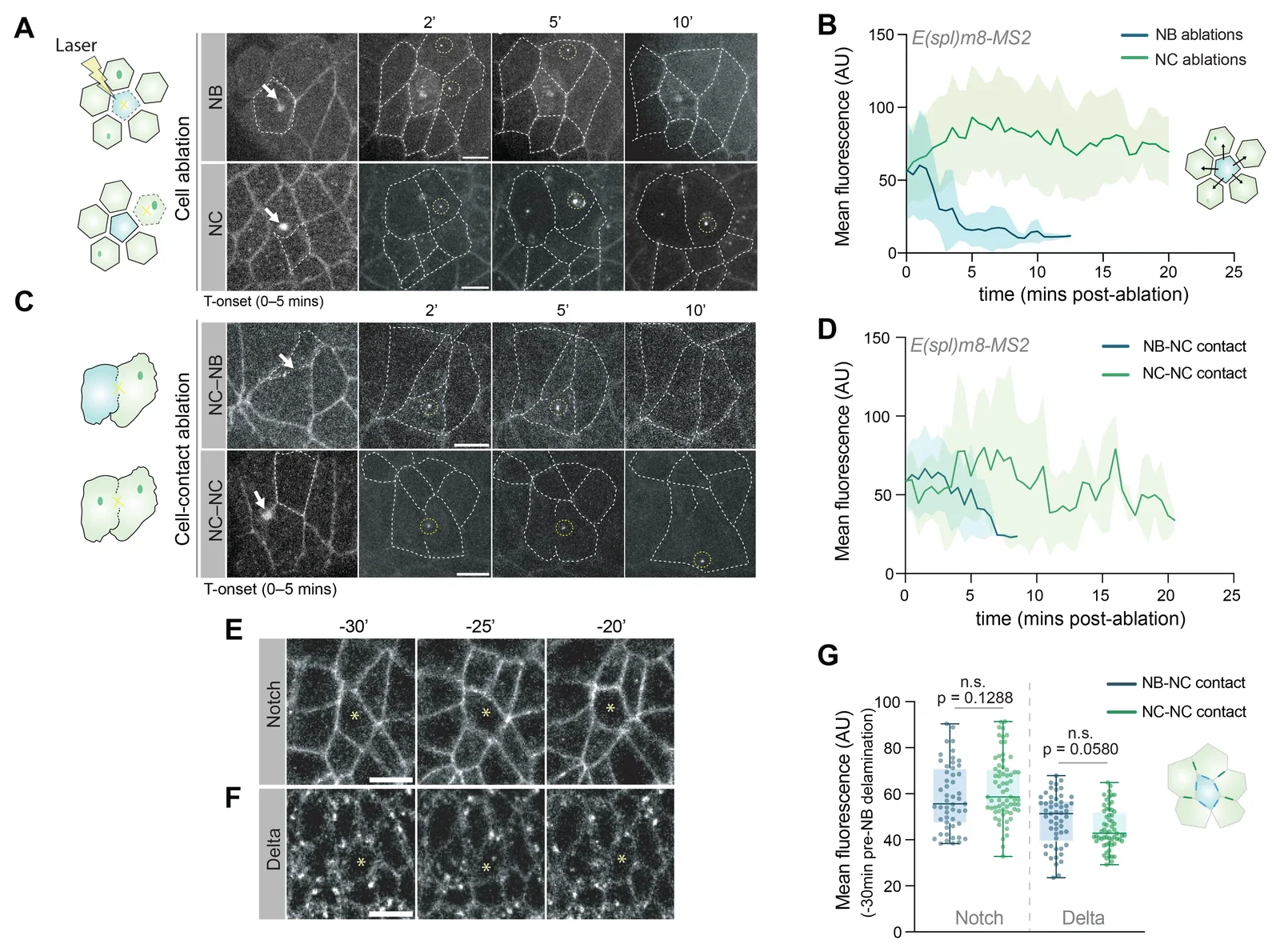
Presumptive Neuroblast (NB) is the primary signal-sending cell within proneural clusters **(A)** Schematic summarising single-cell laser ablations and representative images from each type. White arrows indicate ablated cells; dotted lines outline cell boundaries; yellow circles mark *E(spl)m8-MS2*/MCP transcription foci. NBs are outlined in first panel. **(B)** Mean *E(spl)m8–MS2/MCP* fluorescence traces over time following ablation of NB (blue) or NC (green). Mean duration, NB ablated, 9.00±2.08 mins (N= 16), NC ablated, 16.00±3.56 mins (N= 26). Shaded regions indicate SD. **(C)** Schematic summarising cell-contact laser ablations and representative images from perturbing NB–NC (top) or NC–NC interfaces (bottom) immediately after T-onset (0–5 min). **(D)** Mean *E(spl)m8–MS2/MCP* fluorescence over time following NB–NC (blue) or NC–NC (green) contact ablations. Mean transcription, NB ablated, 6.0±1.7 mins, (N=10) NC ablated, 13.0±3.9 mins (N=10). Shaded regions indicate SD. **(E–F)** Representative images from time-lapse of **(E)** Notch::Halo and **(F)** Delta::mScarlet in neighbour-clusters at indicated times (mins) before NB (yellow asterisk) delamination, -25 is equivalent to T-onset. **(G)** Mean fluorescence intensities of Notch::Halo (left) and Delta::mScarlet (right) at NB-NC (blue) versus NC-NC (green) contacts. Inset schematic indicates regions quantified. Dots, average values for individual NB or NC-contacts. (Notch: N=51 NB, 67 NC contacts; Delta: N=53 NB, 60 NC contacts). See also Video S3 and Table S1.

Ablation of the presumptive NB led to a rapid decline in *E(spl)m8–MS2* intensity in adjacent NCs within 5–6 minutes, demonstrating that the NB provides the signal needed to sustain NC transcription. In contrast, ablating an individual NC did not similarly diminish transcription in adjacent cells (Figure 2A,B). This suggests that NCs do not act as significant sources of signal. Rather, they behave as recipients of NB-derived signalling.

As Notch activation depends on direct ligand–receptor engagement at cell-contacts^17,29^, we next performed spatially targeted cell-contact ablations at the same stage. NB–NC contact ablations led to a rapid decrease in *E(spl)m8–MS2* transcription intensities within ~5 mins. In contrast, ablation of NC–NC interfaces did not (Figure 2C,D; Video S3). Thus, NC-NC interactions do not appear to contribute substantially to levels of Notch-responsive transcription.

The observations establish that the NB is the primary source of signal activating Notch in NCs. To investigate whether this correlates with a difference in ligand and/or receptor expression at NB–NC interfaces we examined levels of endogenously tagged Notch::Halo and Delta::mScarlet. Neither were significantly different between NB-NC and NC-NC junctions during T-onset period (Figure 2E–G). Furthermore, transcription of *neuralised*, the E3 ligase that regulates Delta activity, was similar in presumptive NBs and NCs at T-onset, based on the active transcription site intensities detected by single molecule fluorescent in situ hybridization (Figure S1D,E).

Thus, bias in *E(spl)m8–MS2* transcription cannot be attributed to differences in ligand or receptor levels. Prior observations that *Delta* transcripts are present uniformly and that NB selection can occur under conditions where Notch or Delta are supplied uniformly also support this conclusion^6,30^.

### A bias in apical size prefigures transcriptional onset within clusters

Differences in cell size, contact length and/or contact durations can shape Notch signalling profiles^15^. We therefore asked whether any morphological differences between cells in a neighbour-cluster prefigure the bias in Notch-dependent transcription.

**Figure 3:**
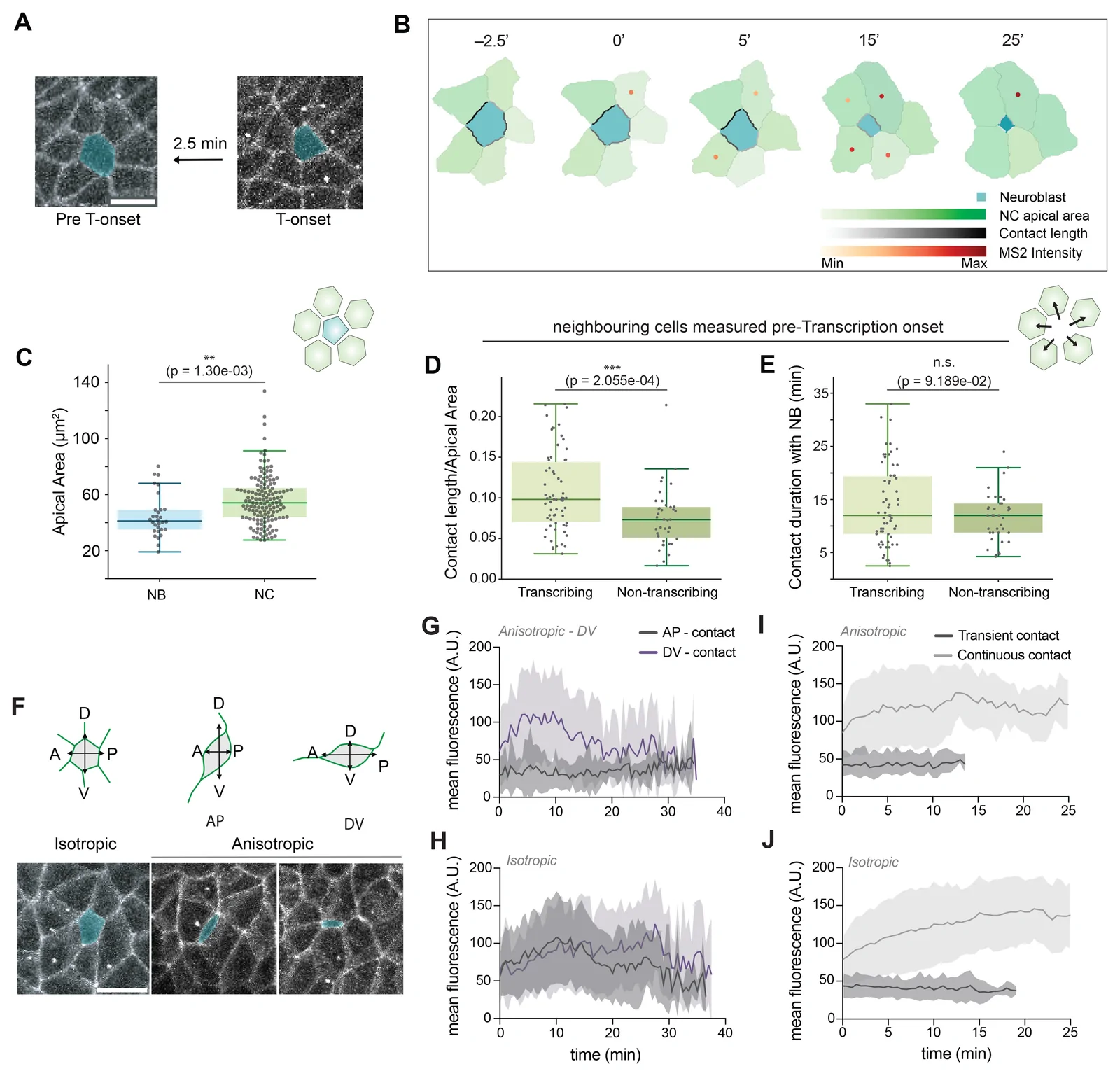
Quantifying apical areas and cell contacts within neighbour-clusters reveals a bias that pre-figures transcription **(A)** Images from a neighbour**-**cluster before transcription onset (Pre T-onset) and at first detectable *E(spl)m8–MS2/MCP* transcription (white puncta; T-onset). Spider::GFP marks cell membranes, presumptive NB is shaded blue. **(B)** Maps illustrating quantified apical area (green scale), contact lengths (black scale) and transcriptional activity (MS2 intensity, red scale) in NCs over time (–2.5’, 0’, 5’, 15’, 25’ mins) for a neighbour-cluster, NB shaded blue. Legend indicates colour scale as labelled. **(C)** Quantification of mean apical areas for presumptive NBs (blue, 44.764±15.283 μm^2^, N=30) versus NCs (green, 56.192±17.873 μm^2^, N=144) over 2.5-minute window before T-onset. **(D)** Mean NB-NC contact lengths during 2.5-minute window before T-onset for NCs that became transcribing (0.107±0.049) or non-transcribing (0.075±0.036). N=66 transcribing, 39 non-transcribing. **(E)** Durations of NB-NC contacts before T-onset in NCs that became transcribing (11.788±4.560 mins) vs non-transcribing (13.780±7.434 mins). N=66 transcribing, 39 non-transcribing. **(F)** Schematic summarising NB delamination geometries with representative images (NB blue shading, Spider::GFP, cell membranes). Isotropic: NB contacts ~5–6 NCs, rosette-like cluster. Anisotropic: NB contacts fewer (~2) NCs, elongated shape aligned along DV or AP axis. **(G–H)** Mean *E(spl)m8–MS2/MCP* fluorescence over time from NCs in DV anisotropic **(G)** and isotropic (**H**) clusters, plotted according to NC position relative to NB, AP (dark grey) or DV (purple), shading indicates SD. N=8 anisotropic, 22 isotropic clusters. **(I–J)** Mean *E(spl)m8–MS2/MCP* fluorescence over time from NCs in continuous (light grey) versus transient (dark grey) contact with NB for anisotropic (**I;** N=15 continuous, 20 transient) and isotropic (**J**, N=69 continuous, 40 transient) clusters. See also Figure S2 and Table S1.

All cells within clusters were segmented and tracked in real-time. Next, apical area, cell-cell contact lengths, durations and *E(spl)m8–MS2* transcriptional dynamics were quantified at single-cell resolution (Figure 3A–B). To determine whether differences preceded T-onset, we first analysed the 2.5-minute time-window prior to T-onset (Figure 3A), beginning just as mesoderm invagination completed (Stage 7). During this interval, cells in each neighbour-cluster maintained a consistent size and all underwent myosin contractions of similar magnitudes (Figure S2A–B)^31^. The overt decrease in NB apical area and change in contractility became evident circa 5’ later when it began to actively ingress. However, the quantitative analysis revealed that the presumptive NB already had, on average, a smaller apical area than other cells in the cluster prior to T-onset (Figure 3C, Figure S2B). Where clusters contained two cells with smaller than average apical areas, the two usually became separated over time and the one remaining within the cluster went on to delaminate (Figure S2C). Thus, the bias in *E(spl)m8–MS2* transcription is preceded by a size differential, with smaller apical area prefiguring the cell that becomes signal-sending and emerges as the NB.

To assess whether cell contact influences *E(spl)m8–MS2* transcription, we quantified the total NB–NC contact duration before T-onset and the mean interface length during the 2.5 min preceding T-onset. Cells that initiated E(spl)m8–MS2 transcription had longer NB–NC interfaces before T-onset than those that did not (Figure 3D), whereas contact duration did not differ significantly (Figure 3E).

Using receiver operating characteristic (ROC) analysis, we asked whether NB-NC contact length, contact duration, or NC apical area could discriminate which cells within a neighbour-cluster were more likely to initiate *E(spl)m8–MS2* transcription (Figure S2D). NB-NC interface length emerged as the strongest individual classifier (area under the ROC curve, AUC = 0.76), NB-NC contact duration showed weak predictive ability (AUC = 0.55) and NC apical area appeared non-predictive (AUC = 0.52~0.5).

To explore combinatorial effects from the different properties, we evaluated joint predictive performance using two supervised classification approaches (logistic regression and random forests), trained using repeated stratified 70/30 train–test splits to assess out-of-sample performance. Multivariate classifiers integrating all features achieved robust predictive performance (best model mean AUC= 0.749±0.067; Figure S2E,F), suggesting they relate to the probability of Notch-activated transcription. Assessing the unique contribution of each predictor using Permutation Feature Importance analysis confirmed that NB–NC interface contact length was the dominant driver, with contact duration making a mild contribution (Figure S2G).

### Sustained contact with presumptive Neuroblast is required for transcriptional activity

Even if NB-NC contact duration is not a sufficient predictor for transcription initiation, a threshold duration may be required. Most NCs that rapidly lost contact, due to rearrangements, failed to initiate transcription. In anisotropic clusters, where the NB acquired an oval shape (oriented along the embryonic AP or DV axis) indicative of differential cell-interface contractions ^22,32^, the NB retained contacts with two flanking cells (Figure 3F). In line with ROC and multivariate classifier analyses, *E(spl)m8–MS2* was only robustly switched on in cells contacting the NB long-axis (Figure 3G, Figure S2H). In clusters where NB ingression was isotropic, contacting cells exhibited similar levels of transcription irrespective of their positions (Figure 3H).

In anisotropic clusters, NCs not adjacent to the NB long-axis rapidly lost contact, retaining tricellular point contacts. We therefore sorted the transcription profiles from both anisotropic and isotropic clusters according to whether NCs had a transient or lasting NB contact. This confirmed that NCs with transient contacts failed to initiate *E(spl)m8–MS2* transcription while those with sustained contact became transcriptionally active (Figure 3I–J).

These results suggest that a threshold size/duration of contact is necessary to generate sufficient signalling to initiate transcription. However, cells that retained contact with the NB long-axis in anisotropic clusters did not achieve a higher mean *E(spl)m8–MS2* transcription intensity than their isotropic counterparts, even though they had longer NB-NC interfaces (Figure 3G–H, Figure S2H). Importantly, this argues against a direct relationship between the length of the NB-interface and the levels of Notch-responsive *E(spl)* transcription per se.

### Mechanical properties and delamination of Neuroblasts shapes the transcriptional response

**Figure 4:**
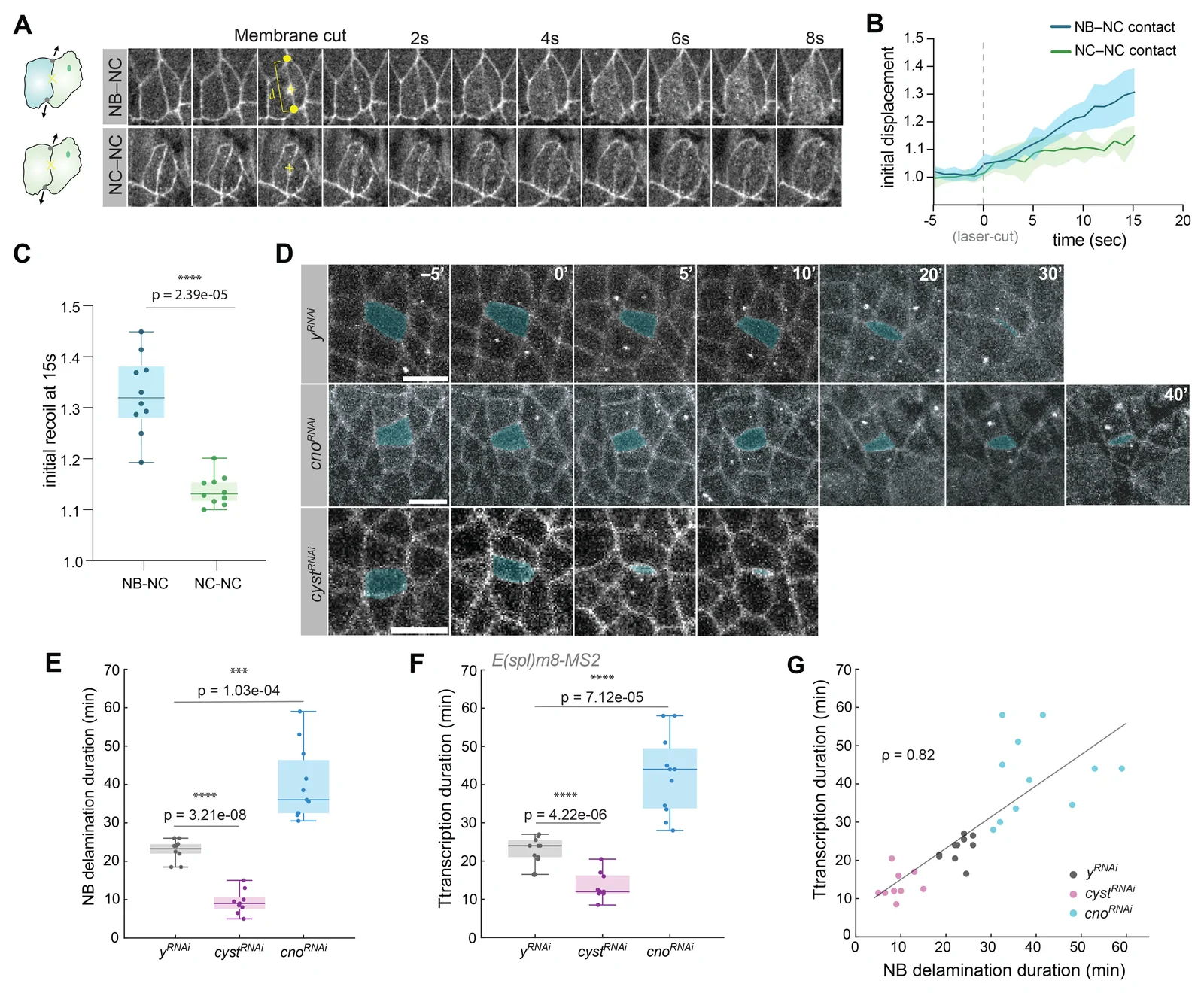
Role of junctional tension and ingression dynamics in modulating *E(spl)m8* transcription profiles **(A)** Schematic and representative time-lapse frames (0–8 s post-laser cut) for each ablation type, NB–NC (top) or NC–NC (bottom), performed immediately after T-onset (0–5 min) in stage 8 *ac-Halo* embryos. Recoil (*d*) of tricellular vertices (yellow dots) flanking each cut membrane was measured to assess junctional tension. **(B)** Quantification of normalized vertex displacement over time for NB–NC contacts (blue) and NC–NC contacts (green) upon ablation. N=10 NB-NC, 10 NC-NC contacts. **(C)** Boxplot of initial recoil amplitudes at 15 sec post-ablation for NB–NC (blue) and NC-NC (green) interfaces. Dots represent individual recoil measurements. N=10 contacts. **(D)** Time-lapse images of NB delamination (cyan) and *E(spl)m8–MS2* transcription (white puncta) in neighbour-clusters from genotypes indicated. Spider::GFP marks cell boundaries. **(E)** Boxplots of delamination duration across genotypes, *y*^*RNAi*^ (control, N=10), *cyst*^*RNAi*^ (N=9) *cno*^*RNAi*^*(*N=11). **(F)** Quantification of cluster transcription duration across genotypes, *y*^*RNAi*^ (control, N=10), *cyst*^*RNAi*^ (N=9) and *cno*^*RNAi*^*(*N=11). **(G)** Correlation between NB delamination and cluster transcription duration across genotypes. Pearson correlation coefficient, 0.82 (p=3.17e-08). See also Figure S3, Video S4 and Table S1.

Since Notch signalling is mechano-sensitive, cell shape and contacts could modulate Notch activation via effects on junctional tension^33,45^. Previous studies have shown that, once NB apical contraction is pronounced, NB-NC contacts are under higher tension than NC-NC contacts^22^. To establish whether there are mechanical differences at earlier stages, we cut individual cell-cell interfaces in *ac-Halo* clusters at the time when *E(spl)m8–MS2* transcription was first detected and measured the initial recoil of tricellular junctions as an indicator of tension along the cell-cell contact^34^ (Figure 4A). We found that NB-NC interfaces displayed greater recoil than NC–NC (Figure 4B,C), indicating they carry higher tension at T-onset.

We next tested consequences of perturbing factors known to regulate mechanical tension in the neuroepithelium^35^. First, we depleted Canoe (Cno), an adaptor protein that links actomyosin to cell junctions and is required for NB delamination to progress normally^22^. In *cno*-RNAi treated embryos, the prolonged period of NB delamination (39.91±9.49 min) was accompanied by an extended period of *E(spl)m8–MS2* transcription (42.45±10.38min; Figure 4E-G; Video S4). Similar effects were seen when embryos were injected with an inhibitor (BAY549) that targets Rho-kinase^36^, whose activity is required for Myosin-II contractility^37^. This treatment prolonged NB delamination (34.06±6 min) and extended the duration of *E(spl)m8–MS2* transcription (38.3±6 min vs 22.6±5 min controls; Figure S3A–C).

Lastly, we depleted the p114 Rho GTPase guanine nucleotide exchange factor encoded by *cysts* (*cyst*), which activates Rho1 at adherens junctions and stabilizes junctional myosin. Its depletion, which leads to more rapid NB ingression (9.39±3.07min; Figure 4D,E; Video S4)^38,39^, reduced the duration of *E(spl)m8–MS2* transcription (13.50±3.64min; Figure 4F,G). The strong correlation between NB delamination period and duration of *E(spl)m8–MS2* transcription (Figure 4G), argues that *E(spl)m8–MS2* transcription is coordinated with NB apical constriction and ingression. Thus, as the NB develops distinct properties from its neighbours, these properties shape signalling to reinforce the differences, which may contribute to resilience in NB selection. For example, only rare defects in NB lineages were detected in *cno* mutant embryos^40^.

### *E(spl)-m8* transcription correlates with morphological changes in NCs

**Figure 5:**
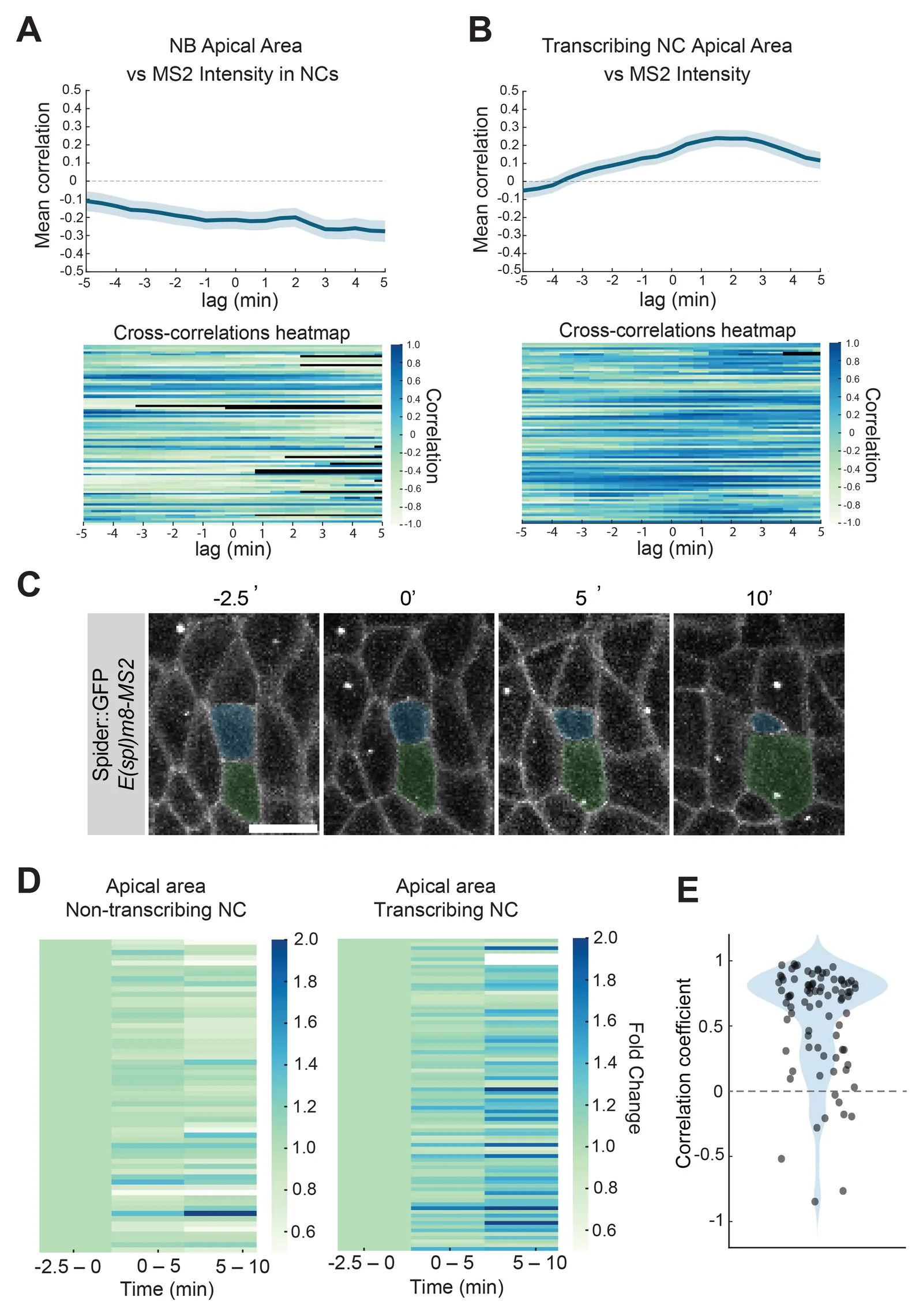
Relationship between *E(spl)m8* transcription levels and cell apical areas. **(A)** Cross-correlation between NB apical areas and *E(spl)m8–MS2/MCP* transcription-levels in NCs; upper plot, mean correlation (over all NCs); bottom heatmaps, individual NC cross-correlations. **(B)** Cross-correlation analysis between NC apical area and *E(spl)m8–MS2/MCP* transcription-levels; upper plot, mean correlation (over all NCs); bottom heatmaps, individual NC cross-correlations. **(C)** Representative images of NC (green) and NB (blue) apical areas before and after T-onset (0’). Transcribing NC apical area increases. **(D)** Heat map indicating apical area fold-change for transcribing and non-transcribing NCs averaged across time-windows: W1, pre-onset (-2.5–0 min, normalized starting area); W2, T-onset (0–5 min); W3, post-onset (5–10 min). Average change: W2: non-transcribing, 0.99±0.16 p=2.88e-1, transcribing 1.10±0.15, p=4.02e-08; W3: non-transcribing 0.95±0.26, p=1.89e-02, transcribing,1.28±0.33, p=6.18e-12; (one-sample t-tests on log2 transformed fold changes). N=60 non-transcribing, 84 transcribing. **(E)** Correlation of NC Apical Areas with area under MS2 curves (a proxy for cumulative mRNA numbers in NC). Violin plot, mean ρ=0.5632±0.4072. See also Table S1.

Given the temporal correlation between *E(spl)m8* transcription and NB delamination, we explored whether transcription levels were related to morphological changes in the NB and/or in NCs. A cross-correlation analysis revealed that NB apical size negatively correlated with *E(spl)m8–MS2* transcription intensity in NCs (Figure 5A). Thus, as the NB progressively constricted and lost apical area, *E(spl)m8–MS2* transcription levels in NCs increased.

In contrast, cross-correlation between *E(spl)m8–MS2* intensities and apical areas of cognate transcribing cells revealed a positive correlation, which maximised at ~2 min positive time delay (Figure 5B). This implies that the increase in apical area follows the increase in *E(spl)m8–MS2* intensity in that cell. An increase in NC apical areas was also detected in previous analyses of cell morphologies during NB ingressions^22,23^. To investigate further, we quantified apical area trajectories of transcribing and non-transcribing neighbours in relation to T-onset using three time-windows (2.5 mins prior to T-onset, 0–5 mins and 5–10 mins after T-onset; Figure 5C,D). Transcribing cells showed a marked increase in apical area following T-onset whereas non-transcribing neighbours (cells that lose NB contact or fail to reach a threshold contact-length for activation) maintained relatively stable apical areas (Figure 5C,D). In addition, the apical area of a transcribing NC and its cumulative *E(spl)m8–MS2* level (area under MS2 curve, a proxy for mRNA numbers) were strongly correlated (Figure 5E). This implies that Notch activation quantitatively impacts on cell morphology, consistent with effects from depleting Delta at this stage^23^.

### Lateral inhibition model incorporating cell perimeter and tension-differences can replicate signalling properties

Our experimental analysis indicates that (i) a bias in NB apical geometry and tension precedes transcription; (ii) NCs transcribing *E(spl)m8* undergo a marked increase in apical perimeter that correlates with transcription levels. These observations prompted us to test whether a modified lateral inhibition framework incorporating differences in apical cell perimeter and tension could account for the observed *E(spl)m8* expression pattern, including the absence of transcription in presumptive NBs.

**Figure 6:**
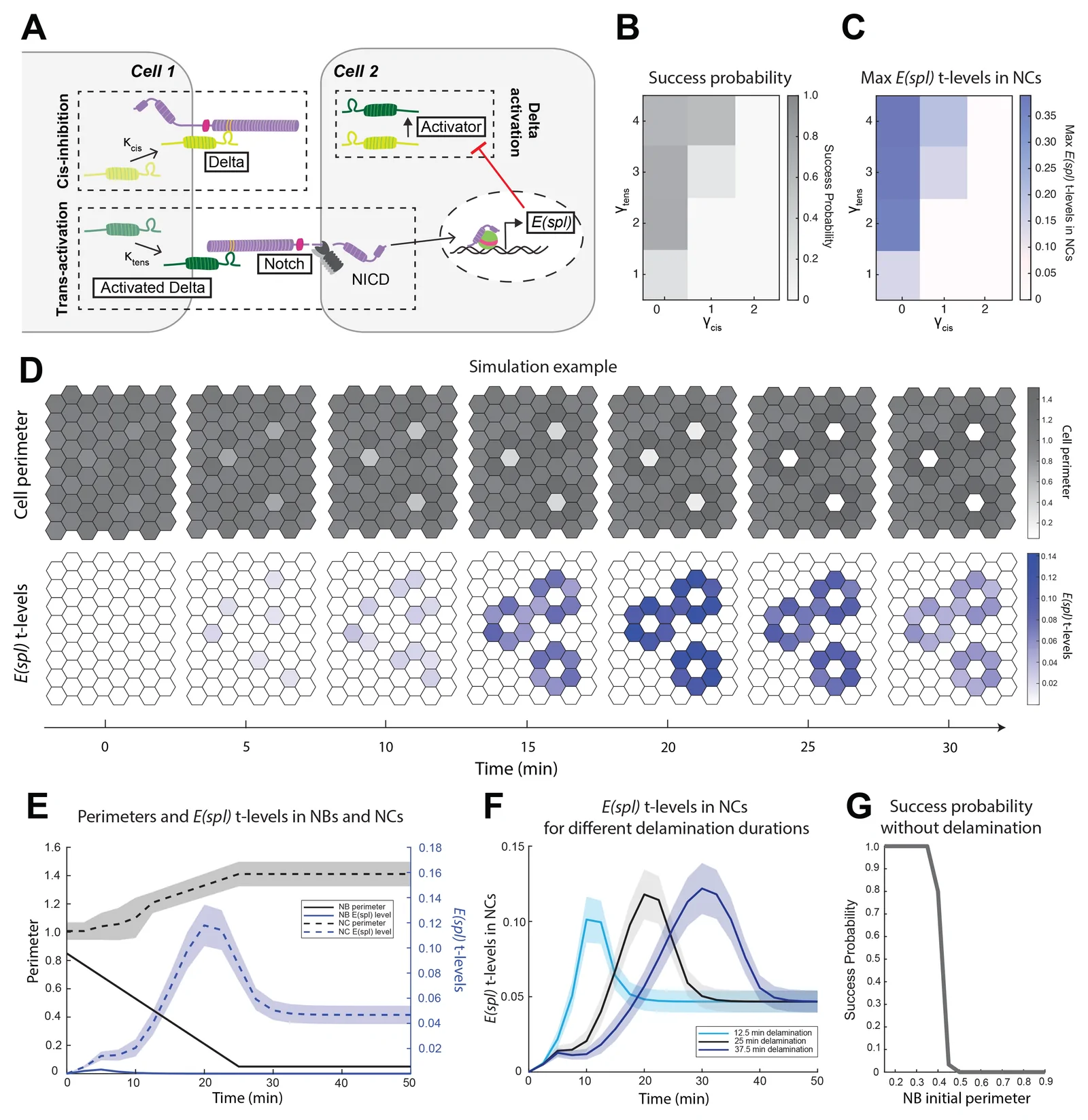
Incorporating morphological features into lateral inhibition model recapitulates in vivo *E(spl)* expression dynamics. **(A)** Schematic of lateral inhibition model, depicting cis-inhibition and transactivation of Notch by Delta. Trans-activation releases NICD, driving *E(spl)* gene transcription (E) leading to Delta inhibition. κ_cis_ scales rate of cis-inhibition, κ_tens_ scales activated Delta, both inversely proportional to cell perimeter. **(B)** Heatmap of simulation success probabilities, averaged over different *E(spl)* detection threshold values and calculated across values of γ_cis_ and γ_tens_, Simulation duration, 50mins, darker shading indicates higher success probability. **(C)** Heatmap of maximum *E(spl)* t-levels in NCs from simulations, averaged over different *E(spl)* detection threshold values and computed across values of γ_cis_ and γ_tens_. **(D)** Representative successful simulation of an 8×8 2D lattice (Video 5), temporal cell-perimeter changes (top, grey scale) and *E(spl)* t-levels (bottom, blue scale). NC perimeter was set to increase once *E(spl)* t-levels exceeded a defined threshold. Parameters used: *E(spl)* detection limit =0.015, initial perimeter for pre-NBs =0.85, for all other cells =1 (±5% noise), γ_cis_=1, γ_tens_=3. Delamination was enforced. **(E)** Quantification of perimeter (grey) and *E(spl)* t-levels (blue) over time for NBs (solid curves) and NCs (dashed curves) from simulation in (D). NB delamination was set to occur over 25 min. **(F)** Effect of NB delamination duration on NC *E(spl)* t-profiles, representative simulations using 12.5 min (light blue), 25 min (grey; same as E), and 37.5 min (purple). Mean transcription durations over random seeds (time taken to reach E steady state): 21.20±0.11min for 12.5min, 33.02±0.07min for 25 min, 44.73±0.08min for 37.5min. **(G)** Simulation success probability for NB initial perimeter values in the absence of delamination. Parameters used: *E(spl)* detection limit =0.012, γ_cis_=1, γ_tens_=3. See also Figure S4, Figure S5 and Video S5.

Because Notch and Delta predominantly localise to apical adherens junctions at this stage^25^, we modified a previously validated two-dimensional lateral inhibition model^14^. In the model, cells express Notch and Delta that can interact in trans, to produce signalling (NICD), or in cis, causing cis-inhibition of receptors and ligands (Figure 6A). To account for contact-dependent signalling, we introduced perimeter-weighted cell– cell connectivity (see Methods S1), which affects the function of Notch and Delta along cell boundaries and we do not pre-suppose differential activator expression as in the original model.

We included two perimeter-dependent parameters to allow mechanical and geometric differences to influence signalling interactions directly (Figure 6A; see Methods S1). The first, a “tension” factor, κ_tens_, based on the hypothesis that higher tension in smaller cells results in higher Delta activity. Support for this assumption includes evidence that mechanical tension on the ligand/receptor bridge is necessary for activating (S2) cleavage *in vivo*^41,51^ and that application of force extends ligand-Notch bond lifetimes^42^, increasing the probability that a receptor can become activated. The second, a “cis-inhibition” factor κ_cis_, based on the hypothesis that cis-inhibition scales inversely with cell perimeter. In a regime where the diameter of the cell contacts exceeds the diffusion-length scale of the ligand^43^ a smaller perimeter would translate to a higher membrane-protein local density, favouring the likelihood of Delta and Notch interacting in cis^44^.

To incorporate the size-change in the delaminating NB and apical expansion of NCs that initiate *E(spl)* transcription, we imposed (i) a time-dependent decrease in NB perimeter and (ii) an increase in perimeter in cells whose *E(spl)* transcription-levels (t-levels) exceeded the detection threshold (based on the positive correlation between accumulated *E(spl)m8-MS2* levels and NC area) (Figure 6D).

To explore how our assumptions and parameters affected lateral-inhibition patterns, we ran simulations for different values of γ_tens_ and γ_cis_, the exponents determining the perimeter dependency of κ_tens_ and κ_cis_. The probability of successful simulations, with no expression in NBs, and maximal *E(spl)* t-levels in NCs (Figure 6B,C; see Methods S1 for success criteria) increased with higher values of γ_tens_ (i.e. stronger dependence of Delta activity on tension). In contrast, increasing γ_cis_ (i.e. stronger dependence of cis-inhibition on perimeter), reduced success probability and peak *E(spl)* t-levels in NCs. However, profiles obtained with γ_cis_>0 included a decrease in *E(spl)* t-levels following their peak, similar to those observed. Under realistic parameter combinations, the model reproduced key features: dynamic and asynchronous *E(spl)* accumulation in NCs and absence of expression in the presumptive NB (Figure 6D,E; Video S5).

We next examined the role of delamination dynamics, by varying the rate at which NB perimeters reduced. This changed the time required to achieve maximal *E(spl)* t*-*levels in NCs in the same direction as we observed in experiments. Prolonged delamination delayed the peak and vice versa (Figure 6F and 4G). In the absence of NB delamination, successful simulations were only obtained when the initial size differential was unrealistically large (NB:other ratio <0.5).

We then asked whether similar outcomes could arise from fully dynamic perimeter behaviour rather than imposed changes. To test this hypothetical scenario, we introduced an ordinary differential equation governing perimeter evolution as a function of *E(spl)* t-levels and active Delta levels (see Star Methods and Methods S1). Additional success criteria were imposed to require NB delamination (see Methods S1) and we assessed whether the experimentally measured initial perimeter differential was sufficient to drive pattern formation under these conditions (Figure S4).

Within a 10 timepoint simulation window (~25 min), recapitulation of experimental behaviour was achieved for an initial perimeter ratio of ≤0.75 (Figure S4A), which closely matches experimentally measured values (Figure 3). Larger initial differentials further increased success probability. In this dynamic model, *E(spl)* t-levels in NCs reached higher amplitudes than in the imposed-perimeter model and activation coincided temporally with NB delamination and with reinforcement of NC perimeter, consistent with experimental observations (Figure S4B,C). However, at longer simulated times, additional cells began expressing *E(spl)* and extra cells underwent delamination (Figure S4D), suggesting that regulatory mechanisms, beyond those in the present model, restrict the outcome at later times. Indeed, in vivo many of the cells undergo mitosis^26^.

As with classical explanations of Notch-mediated lateral inhibition, our model invokes a feedback mechanism that increases Delta activity in the signal-sending cell relative to neighbours. Although no quantitative difference in Delta::mScarlet distribution between NB and NCs was detected at T-onset as discussed above (Figure 2E–G) a change in Delta enrichment appeared subsequently. Once *E(spl)m8–MS2* transcription levels in NCs increased and definitive NB delamination was underway, Delta::mScarlet accumulated in puncta within the NB (Figure S5). These observations are consistent with a model linking the apical cell-cell contacts and tension changes to effects on Delta activity.

Although we acknowledge that our assumptions will require further validations, the simulations illustrate that a lateral inhibition model based on an initial geometric bias can yield the correct profile of *E(spl)* expression, including its exclusion from the NB, when coupled with tension- and cis-inhibition-dependent effects.

## Discussion

Many cell-fate decisions occur concurrently with morphological changes, which are increasingly recognised to play an active role in the decision-making process. For example, mounting evidence argues that Notch signalling can be modulated by global and local mechanical cues^20,45^ as we propose here. Investigating the dynamic properties of cells undergoing Notch signalling during neurogenesis, by measuring *E(spl)m8* transcription in real-time, we demonstrate that NB fate selection is biased from the outset. Our experimental results and mathematical modelling suggest that morphological and mechanical asymmetries help prime the system. Transcriptional feedback then reinforces these differences to establish distinct cell fates.

During neurogenesis, Notch-mediated lateral inhibition is thought to occur among cells with equal neural potential, each initially signalling to its neighbours until small stochastic differences are amplified by transcriptional feedback to specify a single neural precursor^1–3^. Our data challenge this model. We identify a pre-existing bias within proneural clusters that precedes detectable Notch signalling, as measured by *E(spl)m8* transcription. One cell consistently fails to initiate transcription and, as demonstrated by ablation experiments, becomes the signal-sending neuroblast (NB). This occurs without detectable differences in ligand expression, consistent with studies showing that uniform ligand expression is permissive for normal neural precursor selection ^6^. Remarkably, biases in the apical areas already exist in clusters at this “pre-signalling” stage: those cells with smaller apical areas became the signal-producing cell and delaminated as NB. Further studies will be needed to elucidate the underlying mechanisms conferring bias. SoxB and Snail family transcription factors, which are expressed dynamically in the ventral neuroectoderm, are potential candidates^46,47^. Differential forces from germ band extension could also contribute^48,49^.

How could a difference in perimeter and tensions in the presumptive NB confer a bias in signalling that distinguishes it from its neighbours? First, we propose that a smaller perimeter translates to a higher membrane-protein local density, favouring the likelihood of Delta and Notch interacting in cis, making this cell less receptive. Manipulations of Notch and ligand levels have shown that higher relative levels favour cis-inhibition in a manner that may be stoichiometric^44,50^. Second, we propose that higher tension in the presumptive NB increases Delta activity because increased force extends ligand-Notch bond lifetimes^41^ and mechanical tension on the ligand is necessary for the activating S2 receptor cleavage^51^. Indeed, tension heterogeneities modulate Notch activity in several contexts^20,21,45^. Together these properties could render the smaller cell more deaf to signals from its neighbours and more prone to produce a trans-active Delta signal.

To explore whether heterogeneities in tensions and lengths of cell-cell contacts could bring about the observed *E(spl)m8* expression dynamics, we incorporated them into a model of lateral inhibition^14^ introducing two perimeter-dependent parameters based on the reasoning above^12–14^. Initiating the model with the small differences detected experimentally, we could replicate key features of *E(spl)* expression. Thus, our simulations suggest that subtle morphological asymmetries could shape lateral inhibition, as occurs in the chick inner ear^52^, and intestinal stem-cell differentiation^53^, albeit our hypothetical assumptions will require further validations.

These models might initially seem surprising: smaller cells have shorter contacts with their neighbours, yet our results suggest that a longer NB-NC contact predicts transcription initiation. The key is not the absolute contact length, as we find no relationship between contact length and *E(spl)m8-MS2* transcription levels. What likely matters is the relative length of a given NB-NC contact compared to the others made by a NB. If, as our ablation experiments suggest, the NB maintains a higher net level of active Delta, a longer NB-NC interface is likely to reach a critical signalling threshold before shorter ones. Meanwhile, because Notch-Delta signalling is highly sensitive to relative levels and dynamics of ligands and receptors, many interfaces where both are present (such as NC-NCs) will fail to undergo productive signalling due to cis-inhibition^12,13,14^. This can explain why the NB is refractory to signals from NCs and why NCs do not substantially signal to one another, as evidenced by our ablation experiments.

A key feature of neuroblast fate specification is its delamination, a process which normally lasts for circa 20-25 minutes and relies on tension anisotropy between NBs and neighbours^22,23^. The period of *E(spl)* gene transcription in each proneural cluster closely mirrored the delamination time, suggesting the two are coordinated. Furthermore, manipulations that perturb cytoskeletal components involved in regulating tension, such as depletion of *cyst* and *canoe*^22,38,39^, had concomitant effects on delamination times and *E(spl)m8* expression. This argues that the NB and its morphological transitions are important in orchestrating the levels and duration of Notch signalling.

In addition, our observation that cell perimeters increased in proportion to the levels of *E(spl)* transcription argues for a positive reinforcement loop, whereby Notch activity increases apical areas and likely reduces tension in neighbouring cells. Previous studies of cell shapes and tensions during neurogenesis also detected an increase in NC apical area and reduced NC tension^22,23^ which was compromised when Notch activity was inhibited^23^. Together these feedback mechanisms would reinforce the initial heterogeneities. Indeed, it’s been suggested that expansion of the apical domain in retinal epithelial cells augments Notch activation by recruiting activators or diluting the effective concentration of inhibitors^54^. Models incorporating tension-dependent effects on Delta-Notch binding have also been invoked in other contexts where they recapitulate many forms of patterning^55^.

The absence of *E(spl)m8* transcription in the presumptive neuroblast was unexpected. It implies that Notch signalling acts on a pre-existing bias that predisposes one cell to become the predominant source of ligand activity. In our analysis, we found that this corresponded to the cell with the smaller apical area. In a related study, Green and Schweisguth have made a similar observation tracking the fate of cells expressing proneural proteins. Both studies reveal that, during neurogenesis and likely in many other contexts, Notch signalling acts within a landscape that is already primed and serves to re-enforce an established bias. The concept that such bias is manifest in morphological differences that become reinforced through signalling offers a powerful way to integrate signalling with tissue architectures.

## Resource Availability

### Lead contact

Requests for further information and resources should be directed to and will be fulfilled by the lead contact, Sarah Bray (sjb32@cam.ac.uk).

### Material Availability

All unique/stable reagents generated in this study are available from the lead contact without restriction.

## Star Methods

**Table:** 

REAGENT or RESOURCE	SOURCE	IDENTIFIER
Chemicals, peptides, and recombinant proteins		
Rho-kinase inhibitor-BAY549 Azaindole-1	Tocris Bioscience	TC-S 7001
HaloTag® TMR	Promega	G825A
HaloTag® Oregon Green	Promega	G2801
VECTASHIELD Mounting Medium	Vector Laboratories	
Deposited data		
Deposited raw and analyzed data	This paper	https://figshare.com/s/a3665194df21a645f6ff
Code used for analyses and modelling	This paper	https://github.com/crou607/neurogenesis_analysis.git
		
Experimental models: Organisms/strains		

*D. melanogaster*: Spider::GFP: w[*];P{w[+mC]=PTT GB}gish[Spider]	Bloomington Drosophila Stock Center	BDSC_59025
*D.melanogaster*: His2Av-mRFP: w[*];P{w[+mC]=His2Av::mRFP1} II.2	Bloomington Drosophila Stock Center	BDSC_23651
*D. melanogaster*: nos-MCP::GFP: *w; His2Av::mRFP, nos-MCP::eGFP;;*	Bloomington Drosophila Stock Center	BDSC_60340
*D. melanogaster*: *achaete-Halo: w; ac-3kb promoter-7×Halotag/CyO; Spider::GFP/TM6b*	Gift from Prof Yan, (Hong Kong University of Science and Technology)	N/A
*D. melanogaster*: y1 w*; P{mata4-GAL-VP16} 67; P{mata4-GAL-VP16}15	Bloomington Drosophila Stock Center	BDSC_80361
*D. melanogaster*: *E(spl)m7-MS2: +/CyO; E(spl)7-MS2/TM6b* (III)	Santa-Cruz Mateos et al.^24^	
*D. melanogaster*: *E(spl)m8-MS2: +/CyO; E(spl)m8-MS2/TM6b* (III) *(dsRED removed)*	Falo-Sanjuan et al.^25^	
*D. melanogaster*: Delta::mScarlet: *w; Sco/CyO; Delta::mScarlet; TM6b*	Boukhatmi et al.^56^	
*D. melanogaster*: Notch::Halo: Notch{2xHalo} */FM7a*	C. Santa-Cruz Mateos (U of Cambridge)	
*D. melanogaster*: Cyst^RNAi^ VALIUM 20 (II): y[1] sc[*] v[1] sev[21]; P{y[+t7.7] v[+t1.8]=TRiP.HMS01750}attP40	Bloomington Drosophila Stock Center	BDSC_38292
*D. melanogaster*: Cno^RNAi^ VALIUM 22 (II): y[1] sc[*] v[1] sev[21]; P{y[+t7.7] v[+t1.8]=TRiP.GL00633}attP40	Bloomington Drosophila Stock Center	BDSC_38194
*D. melanogaster*: *y^RNAi^* (control) VALIUM20: y[1] sc[*] v[1] sev[21]; P{y[+t7.7] v[+t1.8]=TRiP.HMC04284}attP40	Bloomington Drosophila Stock Center	BDSC_55988

Oligonucleotides		
Stellaris Probe SMFish *neuralised* probes	Bioscience Technologies	N/A
Software and algorithms		
Fiji-Image J	Schindelin et al.^57^	N/A
Tissue analyzer	Aigouy et al.^58^	N/A
Trackmate-Fiji Plugin	Tinevez et al. ^59^	N/A
MATLAB	MathWorks	N/A

### Experimental model and study participant details

*Drosophila melanogaster*. Flies were grown and maintained on food consisting of glucose 76 g/l, cornmeal flour 69 g/l, yeast 15 g/l, agar 4.5 g/l, and methylparaben 2.5 ml/l. *Drosophila* stocks and crosses were raised and maintained at 25°C unless stated otherwise. Embryos were collected on apple juice agar plates with yeast paste.

### Fly strains and Genetics

Details of *Drosophila melanogaster* strains used in this study are listed in Supplementary Table 1, including those obtained from the Bloomington *Drosophila* Stock Center.

To monitor transcription from the endogenous *E(spl)-C* locus, E(spl)*m8-MS2*^25^ *or E(spl)/m7-MS2*^24^ lines were crossed to *His2Av::RFP, nos-MCP::GFP; Spider::GFP* to generate embryos of the genotype: *His2Av::RFP, nos-MCP::GFP* (II); *E(spl)m8–MS2* / *Spider::GFP* (III). (Figure 1C).

To label proneural clusters in the neurectoderm, *achaete-Halo (ac-Halo,); Spider::GFP* males were crossed to *His2Av::RFP, nos-MCP::GFP; E(spl)m8–MS2* females, generating embryos of genotype: *ac-Halo / nos-MCP::GFP (II); Spider::GFP / E(spl)m8–MS2 (III)* (Figure S1A). *ac-Halo* (gift of Yan Yan, Hong Kong University) consists of the ac promoter and 3kb upstream sequences driving 7xHalo7::CAAX (from Addgene ID 87647).

To analyse Notch and Delta protein levels along membranes, Notch{2xHalo} */FM7a; +/Cyo; +/TM6b* and *+/Cyo; Delta::mScarlet/ Delta::mScarlet* lines were used. (Figure 2E–F)

*cyst*^*RNAi*^, *cno*^*RNAi*^, or *y*^*RNAi*^ (control) were expressed maternally using the *mat-αTub-GAL4* driver (*mat-Gal4*). *mat-GAL4; Spider::GFP* females were crossed to *UAS-(cyst / cno / y)*^*RNAi*^; *nos-MCP::GFP, E(spl)m8–MS2* (recombined on the *III*^*rd*^ chromosome) males to obtain embryos of the genotype: *mat-GAL4 / UAS-(cyst / cno / y)*^*RNAi*^ *(II); Spider::GFP / nos-MCP::GFP, E(spl)m8–MS2 (III)*. (Figure 4D)

For experiments analysing Delta dynamics, males carrying *Delta::mScarlet (III)* were crossed to *nos-MCP::GFP; E(spl)m8–MS2* females. (Figure S5 A)

All crosses were maintained under standard conditions, and embryos were collected and imaged at the indicated developmental stages.

### Method details

#### Mounting and live imaging of *Drosophila* embryos

0–2h, stage 6 embryos were collected on apple juice agar plates. Embryos were dechorionated using bleach and mounted onto 35-mm poly-D-lysine-coated glass bottom dishes (Mattek, P35GC-1.5-10-C). For all recordings (unless specified), embryos were oriented with their ventral/ventrolateral side facing the coverslip and submerged in 1x PBS.

Imaging was performed on a Leica SP8 confocal microscope (unless stated otherwise) using a 40x apochromatic, oil immersion objective (NA 1.3). Spider-GFP and nos-*MCP::GFP; E(spl)m8–MS2* detection were carried out using identical settings across experiments (except laser ablation experiments) and resolved based on their distinct localization within cells: A 40 mW 488-nm argon laser, and two hybrid GaAsP detectors, 8-bit, 600 Hz scanning speed, and a pinhole set to 4-Airy units. For imaging neurectodermal clusters, the following settings were used: 4% 488-nm laser power, 2x zoom, image size 512 × 512 pixel (0.43 μm/pixel), 1μm optical sections of 20–25 z-stacks, and a temporal resolution of 30s per volume. His2Av-RFP or HaloTag® TMR injected embryos were imaged with an additional 2%, 561-nm laser, image size 512 × 512 pixels resolution (0.43 μm/pixel). All scale bars in the figures are 10µM unless otherwise indicated.

#### Embryo injections

HaloTag ligands: To image *ac-Halo* and Notch::Halo, blastoderm stage embryos were collected and oriented ventral side facing the coverslip coated with mounting glue and were covered with halocarbon oil (Voltalef 10s). 0.25 mM of HaloTag® TMR (Promega G825A) or HaloTag® Ore-Green Promega G2801) ligands diluted in injection buffer (180 mM NaCl, 10 mM HEPES [pH 7.2], 5 mM KCl, 1 mM MgCl2) was injected into the perivitelline space of the embryos.

Rho-Kinase inhibitor: Embryos were injected with 2mM ROCK-inhibitor (BAY-549, Tocris Bioscience, TC-S 7001) or DMSO as control. To avoid interfering with ventral furrow formation, Rho-kinase inhibitor was injected into staged embryos immediately after the furrow had closed (early stage 7)

#### Laser ablations

Embryos expressing *ac-Halo, Spider::GFP* and *nos-MCP::GFP; E(spl)m8–MS2* were mounted on the coverslip to ablate single cell or precise cell-contacts. Gastrulation stage embryos were injected with HaloTag®TMR (Promega) 10mins before imaging. Halo was distributed throughout the cell and gradually accumulated to a higher level at juxta-membrane regions due to fusion with C-terminal CAAX motif. *ac-Halo* proneural clusters were located using TMR fluorescence and ablations were performed as soon as a transcription foci appeared in one cell of the cluster. *ac-Halo* positive cells in direct contact with the transcribing cell were targeted according to their position.

#### Optical arrangement of Photoablation unit

The imaging was performed on a Nikon Ti2 inverted-photomanipulation system. A confocal spinning disk system (CrestOptics X-light V3) is connected to the microscope. The confocal system is mainly composed of LDI-7 laser diode Illuminator (89 North Oxxius Inc), a lenslet array, a moveable spinning disk, two cameras (Photometrics Prime 95B), a 100X oil objective (Nikon CFI Plan-Apochromatic DM Lambda 100X Oil NA/1.45). The custom developed photoablation unit is composed of a femtosecond laser (Spectra-physics Mai Tai Deep Sea Ti:Sapphire HP), a polarizing beamsplitter, a Pockels cell (Lambda M350-105-BK-02), a beam expander, a spatial light modulator (Meadowlark 1920 × 1200). The ablation light is emitted from the Ti:Sapphire laser, polarized to plane waves, fast switch on and off by the Pockels cell and expanded to the desired beam size. The collimated beam is projected to the spatial light modulator for reshaping the beam profile to a tight spot, which is relayed to the image plane for ablation (<1 µm in diameter).

In a typical laser ablation experiment on *Drosophila* embryos, the laser wavelength was tuned to 900 nm to match 2-Photon absorption of GFP. Once the ablation site was precisely identified, the shutter was open for 15ms and closed for 20ms, 2 cycles for precise contact-ablations, 3 cycles for NB/NC ablations. The laser output power was set to result in 51 mW for precise contact ablations and 92 mW for NB/NC ablations on the image plane. The ablation process was controlled by a custom-developed LabVIEW (National Instruments) software, while the confocal system was controlled by MetaMorph (Molecular Devices) to continuously image the ablation and subsequent recoil process. Ablation was achieved by focusing the laser onto a single plane. For recoil measurements, single plane images were acquired immediately after ablation at the framerate of 1s/frame for 60s. For dynamic MS2-foci tracking, imaging was switched to acquire z-stacks with 30s/volume for 30 mins.

#### Single molecule in situ hybridization

Custom smFISH probe sets for *neur* gene were designed with Stellaris Probe Designer (Bioscience Technologies). Stage 6–8 embryos were collected on apple juice agar plates and dechorionated in 50% bleach for 1–2 minutes. After thorough washing alternating between ddH_2_O and embryo wash buffer, embryos were transferred to a scintillation vial containing 10 mL fixation solution (0.5 mL nuclease-free water; 0.5 mL 10X Phosphate Buffered Saline (PBS), RNase-free; 4 mL 10% ultra-pure formaldehyde; 5 mL Heptane) and fixed on an orbital shaker 30 minutes. The aqueous phase was removed and replaced with methanol and the vials were shaken vigorously for 30 seconds to devitellinise embryos. Devitellinised embryos settled to the bottom were collected, washed three times in methanol, and stored at −20°C until use.

For hybridisation, embryos were rehydrated through a methanol series (75%, 50%, 25% methanol in PBT) and washed three times in PBT (10 minutes each). Embryos were then added into Wash Buffer A via a 50:50 PBT:Wash Buffer A step followed by two washes in Wash Buffer A (5 minutes each), before pre-incubation in Hybridization Buffer at 37°C for 2 hours. Stellaris probe mixtures were prepared at 50 nM in Hybridization Buffer and hybridised overnight (~14 hours) at 37°C in the dark. Following hybridisation, embryos were washed in pre-warmed Hybridization Buffer (30 minutes, 37°C) then three times in pre-warmed Wash Buffer A (15 minutes each, 37°C) in the dark, followed by a room-temperature Wash Buffer A wash and three PBT washes (10 minutes each). Embryos were mounted in Vectashield mounting medium with DAPI and cured flat in the dark prior to imaging.

#### Quantification and Statistical Analysis:

For details of sample sizes and statistical tests used for each experiment and analysis please see Supplementary Table S1.

#### Cell segmentation and tracking

All image processing and quantitative analyses were performed using Fiji (ImageJ) together with MATLAB (Mathworks) or GraphPad-Prism. Cells were segmented and tracked and measured using the Tissue Analyzer integrated in Fiji^58^ Spider-GFP signal was used to segment cell boundaries and track apical areas, dynamic changes in cell–cell contact lengths and neighbour exchange within proneural clusters. Individual clusters were tracked starting at least 2.5 mins pre-transcription to 10 mins after apical NB delamination. NB and NCs were identified based on delaminating cells and presence of *E(spl)m8–MS2* dependent transcription and were backtracked to measure apical area, contact length and contact durations which were then plotted using a custom MATLAB script.

#### Detection and quantification of transcription foci

Nascent transcription spots from *E(spl)m8–MS2 MCP::GFP* and *E(spl)m7–MS2 MCP::GFP* were tracked using the TrackMate plugin integrated in Fiji^59^. A LoG detector was applied with parameters optimised for the MS2/MCP::GFP foci size (normalized to the ROI diameter) and signal-to-noise characteristics and the mean-intensities of *MCP:GFP* spots were collected. Only transcription foci persisting for ≥ 2 consecutive frames were considered as transcription events. Final MS2/MCP::GFP intensities represent background-subtracted mean fluorescence values in clusters aligned to time 0 (i.e., detection of the first transcription foci to the appearance of last within a cluster).

Measurements included: Cluster transcription duration, time between appearance of first MS2 spot and disappearance of last spot in each cluster; Delamination duration, time between transcription onset and NB disappearance from plane.

#### Combined analysis

Cell perimeter, contact-length, and MS2/MCP::GFP spot tracking obtained from TrackMate were combined using a custom MATLAB script to enable cluster-by-cluster analysis. This allowed simultaneous quantification of cell apical area, NB–interface contact length, and transcription levels over time.

For apical area comparisons shown in Figure 3C, the mean apical area of NBs and NCs was calculated over a 2.5 min window preceding cluster transcription onset. Onset was defined as the time when the first MS2/MCP::GFP puncta appeared within each cluster.

Contact length comparisons shown in Figure 3D was calculated as mean NB–NC contact lengths (normalised by NC area at each timepoint) during the 2.5 min window preceding the transcription start point. For transcribing NCs, this point corresponded to the time when the MS2/MCP::GFP puncta first appeared in that cell. For non-transcribing NCs, a pseudo-transcription start point was defined as the median transcription start time of the cluster.

Durations of NB–NC contacts shown in Figure 3E were measured from the time when the NC and NB first came into contact until the transcription start point of each transcribing NC, or the pseudo-transcription start point for non-transcribing NCs.

#### Measurement of Notch and Delta intensities

Notch::Halo (Oregon Green ligand) and Delta::mScarlet intensities were measured in early-clusters approximately 10 minutes prior to the continuous decrease in the apical-area of the NB. Timing was based on (i) ventral closure and (ii) precisely staging NB delamination and back-tracking. Measurements were made at NB-NC or NC-NC contacts and normalized by subtracting background.

Delta dynamics were analyzed at later times in embryos expressing Delta::mScarlet and *nos-MCP::GFP*; *E(spl)m8–MS2*. NCs were identified by the appearance of MS2/MCP::GFP puncta and cytoplasmic intensities from vesicular Delta were measured in NBs and NCs Absolute intensity values were then normalized by subtracting the background.

#### Recoil measurements

Junctional tension was measured as a proxy of the initial recoil dynamics following targeted laser ablation of individual cell-cell contacts. For each ablation event, continuous displacement of tricellular nodes at both ends of the junction were tracked using the line tool in Fiji. Displacement between each node was measured relative to the initial, pre-ablation distance between the nodes. Displacement trajectories were plotted for the first 15 sec following ablation and across multiple clusters to capture the instantaneous response at ablated contacts. Initial recoil at 15 sec was measured as a derivative of displacement over time.

#### Cross-correlation analysis

Cross-correlation analyses (Figure 5A,B) were carried out using a custom MATLAB script, applying both negative (shifting MS2 curves backward in time) and positive (shifting MS2 curves forward in time) time lags.

For each time lag, the correlation between each shifted MS2 curve and its corresponding NB apical area (Figure 5A) or transcribing NC apical area (Figure 5B) was calculated (heatmaps in the bottom panels), and the average correlation across all tracks was then computed (curves in the top panels).

#### Area fold-change analysis

Fold-change apical area analysis (Figure 5C) was calculated for transcribing and non-transcribing NCs across two time windows, 0–5 min and 5–10 min after transcription onset, relative to their mean starting area during the −2.5–0 min window.

For non-transcribing NCs, the analysis was performed relative to cluster onset (the first time an MS2 spot appeared in the cluster) and continued for as long as the cluster persisted, irrespective of the NC’s contact with the NB.

For transcribing NCs, the analysis was performed relative to the transcription start point in that NC and continued for as long as transcription persisted in that cell, irrespective of NB contact.

#### Modelling

We adapted the lateral inhibition model described in Troost *et al*^14^ to investigate how cell geometry and mechanical properties influence signalling dynamics. In contrast to the original framework, we removed the requirement for a spatial activator gradient and instead implemented a perimeter-weighted cell–cell connectivity matrix. This modification allows the strength of interactions between neighbouring cells to depend on the extent of their shared boundary, reflecting the function of Notch and Delta along cell membranes.

To incorporate potential mechanical effects on signalling, we introduced two additional parameters which scale inversely with cell perimeter. The first parameter, κ_tens_, represents a “tension” factor that scales the amount of activated Delta available for trans-activation of neighbouring Notch receptors. The second parameter, κ_cis_, is a “cis-inhibition” scaling factor (Figure 6A; see Methods S1). These modifications allow cell geometry to modulate signalling interactions directly within the model.

The full mathematical formulation of the model is provided in Methods S1. This includes the reaction scheme corresponding to the processes illustrated in Figure 6A, the derivation of the governing ordinary differential equations, and the quasi steady-state assumptions used to simplify intermediate complexes. The Methods S1 also describes the simulation framework, initial conditions, and parameter values used (and see Table S2).

For analysis shown in Figure 6, the final set of non-dimensionalised equations used is as follows:

(1) $$\frac{dd_{i}}{dt}=\beta_{d}+\alpha^{-}d_{i}^{A}-\alpha^{+}A_{i}d_{i}-\left(1+K_{c,i}n_{i}\right)d_{i}$$

(2) $$\frac{dd_{i}^{A}}{dt}=\alpha^{+}A_{i}d_{i}-\left(\alpha^{-}+1+K_{t}{\langle n_{j}\rangle }_{\text{i}}+K_{c,i}n_{i}\right)d_{i}^{A}$$

(3) $$\frac{dn_{i}}{dt}=\beta_{n}-n_{i}-K_{t}n_{i}{\langle d_{j}^{A}\rangle }_{\text{i}}-K_{c,i}\left(d_{i}+d_{i}^{A}\right)n_{i}$$

(4) $$\frac{dE_{i}}{dt}=\frac{\beta_{E}{\left(\frac{K_{t}}{T_{s}}n_{i}{\langle d_{j}^{A}\rangle }_{\text{i}}\right)}^{c_{s}}}{1+{\left(\frac{K_{t}}{T_{s}}n_{i}{\langle d_{j}^{A}\rangle }_{\text{i}}\right)}^{c_{s}}}-E_{i}$$

(5) $$\frac{dA_{i}}{dt}=\frac{\beta_{A}}{1+{\left(\frac{E_{i}}{T_{E}}\right)}^{c_{E}}}-A_{i}$$

where $d_{i},d_{i}^{A},n_{i},E_{i},A_{i}$ are levels of Delta, activated Delta, Notch, *E(spl)* and activator in cell i respectively, ${\langle d_{j}^{A}\rangle }_{\text{i}}$and ${\langle n_{j}\rangle }_{\text{i}}$are sum of activated Delta and Notch, respectively, from cells j on the boundaries with cell i,

*β*_*d*_, *β*_*n*_, *β*_*E*_, *β*_*A*_ are expression rates of Delta, Notch, *E(spl)* and activator respectively,

*α* and *α*^-^ are the rates of Delta activation by Activator and de-activation respectively,

*K*_*c,i*_ is the rate of cis-inhibition for cell i,

*K*_*t*_ is the rate of trans-activation,

*T*_*s*_ and *T*_*E*_ are the Hill half occupation levels associated with Notch promoting *E(spl)* expression and *E(spl)* inhibiting activator expression respectively, while c_*S*_ and c_*E*_ are the respective Hill coefficients.

The following perimeter rule was set to account for NB delamination and transcribing NC apical area reinforcement:

(6) $$p_{i}(t)=(1-s(t))p_{0,i}+s(t)p_{target\;,i}$$

where,

(7) $$s(t)=\{\begin{array}{cc} 0 & t<t_{0}\\ \frac{t-t_{0}}{\tau } & t_{0}\leq t<t_{0}+\tau \\ 1 & t\geq t_{0}+\tau \end{array}$$

with τ the delamination duration and

(8) $$p_{target\;,i}=\{\begin{array}{cc} 0.05 & \;for\;NB\\ 1.4p_{0,i} & \;if\;E_{i}>{\text{L}}_{\text{D}}\\ p_{0,i} & \;otherwise\; \end{array}$$

where *p*_*i*_ is the perimeter of cell i (with *p*_0,*i*_ *as* the initial perimeter),

*τ* is the delamination duration and *t*_0_ the timepoint when this rule is triggered and L_D_ is *E(spl)* detection limit.

For the dynamic perimeter model (Figure S4), ODEs (1) – (5) were used together with a perimeter ode:

(9) $$\frac{dp_{i}}{dt}=-\kappa_{p}\frac{d_{A,i}^{h}}{\theta_{\delta }^{h}+d_{A,i}^{h}}+\frac{E_{i}^{m}}{\theta_{E}^{m}+E_{i}^{m}}$$

where *k*_*p*_ is the ratio of Activated Delta and *E(spl)* Hill function prefactors respectively, *θ_δ_* and *θ_E_* are the Hill activation thresholds associated with Delta activity inhibiting and *E(spl)* promoting perimeter respectively, while *h* and *m* and the respective Hill coefficients.

The equations are solved numerically in MATLAB using a standard ODE solver.

#### Descriptive statistics and statistical tests

Data are expressed as the mean ± SD, and error bars in graphs represent SD. For all boxplots, the line across the box represents the median, the top and bottom edges correspond to the upper and lower quartiles, respectively, and whiskers extend to 1.5× the interquartile range. Dots represent individual measurements.

For statistical tests involving two samples, two sample t tests (if samples were normal) and Mann-Whitney U tests (if samples were not normal) were performed. Normality of the samples was assessed using Q-Q plots and Shapiro-Wilk tests. Where two samples were compared, equality of variance was also assessed with Bartlett’s test (if samples were normal) and Levene’s test (if samples were not normal). In all cases significance is presented as follows: * p < 0.05, ** p < 0.01, *** p < 0.001, **** p < 0.0001 and unless stated otherwise in the legend, was evaluated with two sample t tests. Full details of sample numbers and of all statistical tests performed are provided in Table S1.

## Supplementary Material

Supplemental Information

Supplemental Methods S1

Supplementary videos

## Data Availability

All Data have been deposited at FigShare and are publicly available as of the date of publication at https://figshare.com/s/a3665194df21a645f6ff All original code has been deposited at Zenodo and is publicly available at DOI 10.5281/zenodo.20612238 as of the date of publication. Any additional information required to reanalyze the data reported in this work paper is available from the Lead Contact upon request.
