# Supplementary material for: Notch mediated lateral inhibition is shaped by morphological differences to reinforce bias toward signal-sending or receiving roles
Source: Curr Biol. Author manuscript; Available in PMC 2026 Sep 12. (PMC7619482; doi:10.1016/j.cub.2026.07.041)
Supplement: Supplementary file 1 [file EMS217835-supplement-Supplemental_Information.pdf]

Current Biology, Volume *36*

Supplemental Information

Notch-mediated lateral inhibition is shaped by morphological differences to reinforce bias toward signal-sending or -receiving roles

Prachi Richa, Charalambos Roussos, Chengxi Zhu, Martin O. Lenz, Shahar Kasirer, David Sprinzak, and Sarah J. Bray

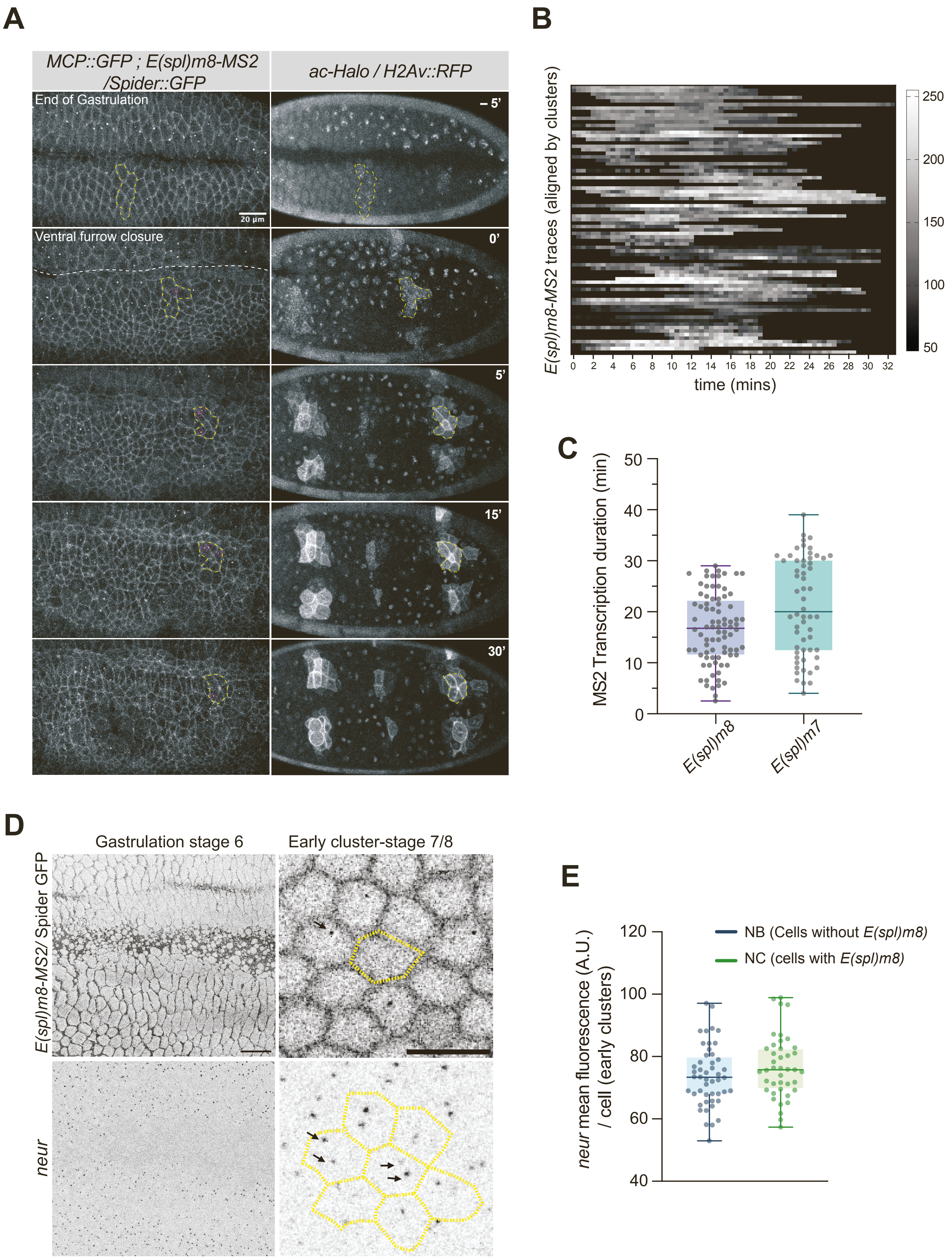
A
MCP::GFP ; E(spl)m8-MS2 /Spider::GFP
ac-Halo / H2Av::RFP
End of Gastrulation
– 5'
20 µm
Ventral furrow closure
0'
5'
15'
30'
B
E(spl)m8-MS2 traces (aligned by clusters)
time (mins)
0 2 4 6 8 10 12 14 16 18 20 22 24 26 28 30 32
250
200
150
100
50
C
MS2 Transcription duration (min)
0 10 20 30 40 50
E(spl)m8
E(spl)m7
D
Gastrulation stage 6
Early cluster-stage 7/8
E(spl)m8-MS2/ Spider GFP
neur
E
neur mean fluorescence (A.U.) / cell (early clusters)
40 60 80 100 120
NB (Cells without E(spl)m8)
NC (cells with E(spl)m8)

Figure S1. Spatiotemporal coordination of cluster transcription. Related to Figure 1.

(A) Time-lapse images of embryo expressing *Achaete-Halo* reporter to mark a subset of proneural clusters (right panel) together with *Spider-GFP* (left panel) to visualise cell membrane, *E(spl)m8–MS2* transcription (bright foci; left panel) and nuclear *H2Av-RFP* (right). *Achaete-Halo* can first be detected as ventral furrow completes closure, labelled t=0, and becomes stronger over time. Scale bar, 20 µm.

(B) Onset of *E(spl)m8–MS2* occurs asynchronously within proneural clusters. Heatmap in which each horizontal row corresponds to *E(spl)m8–MS2* fluorescence intensity in a single nucleus, aligned to the transcription onset within each cluster, N= 30 clusters.

(C) Transcription durations of individual *E(spl)m7–MS2* and *E(spl)m8–MS2* traces (all traces aligned to their onset). Boxplot shows median and interquartile range, dots represent individual measurements corresponding to a transcriptionally active NC. Both genes exhibit comparable on-durations: *m8*, 16 ± 7 min; *m7*, 21 ± 8 min (mean ± SD).

(D) smFISH against neuralized (*neur*) mRNA (bottom panels) in embryos expressing *E(spl)m8–MS2*/Spider::GFP (top panels) at gastrulation stage 6 (left) and early cluster stage 7/8 (right). At stage 6, *neur* transcripts are distributed broadly and uniformly across the neuroectoderm. At stage 7/8, individual *neur* transcripts (arrows) are detectable in all cells within proneural clusters (yellow outline indicates NB in top and cluster in bottom panel). Scale bars, 10 µm.

(E) Quantification of mean *neur* smFISH fluorescence per active transcription site per cell in early cluster-stage embryos, comparing prospective NBs (cells lacking *E(spl)m8* foci, blue) and NCs (cells with *E(spl)m8* foci, green). Each dot represents fluorescence intensities from *neur* foci averaged per cell and normalised to the cytoplasmic background. No significant difference in *neur* transcript levels is detected between the cells within clusters (N= 48 NB, 42 NC cells; p = 0.1703, n.s.).

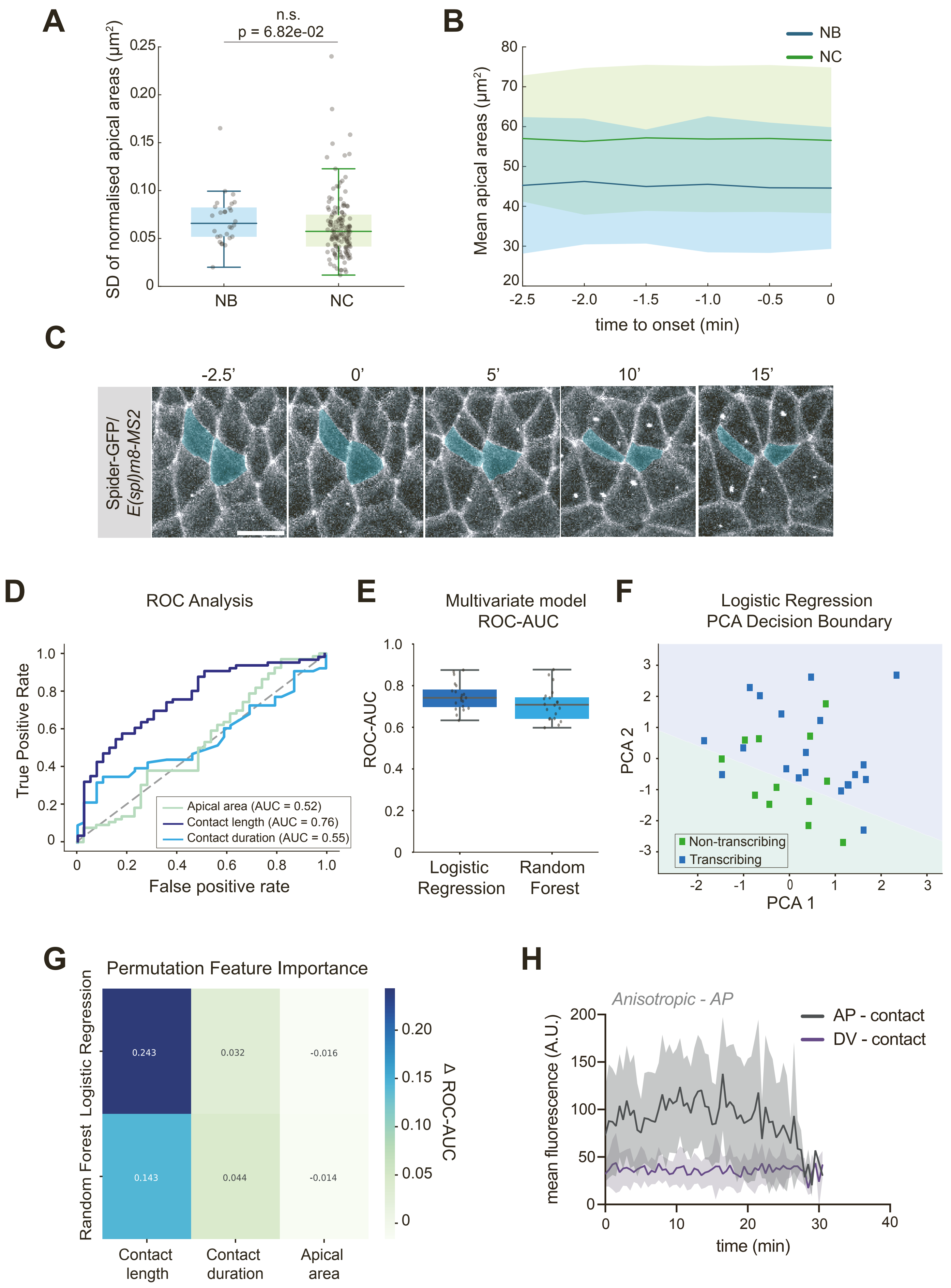

A
n.s.
p = 6.82e-02
SD of normalised apical areas (µm²)
0.25
0.20
0.15
0.10
0.05
0
NB
NC
B
NB
NC
Mean apical areas (µm²)
80
70
60
50
40
30
20
-2.5
-2.0
-1.5
-1.0
-0.5
0
time to onset (min)
C
-2.5'
0'
5'
10'
15'
Spider-GFP/
E(spl)m8-MS2
D
ROC Analysis
True Positive Rate
1.0
0.8
0.6
0.4
0.2
0
0.2
0.4
0.6
0.8
1.0
Apical area (AUC = 0.52)
Contact length (AUC = 0.76)
Contact duration (AUC = 0.55)
False positive rate
E
Multivariate model
ROC-AUC
1.0
0.8
0.6
0.4
0.2
0
Logistic
Regression
Random
Forest
F
Logistic Regression
PCA Decision Boundary
PCA 2
3
2
1
0
-1
-2
-3
Non-transcribing
Transcribing
-2
-1
0
1
2
3
PCA 1
G
Permutation Feature Importance
Logistic Regression
Random Forest
0.243
0.032
-0.016
0.143
0.044
-0.014
Contact
length
Contact
duration
Apical
area
0.20
0.15
0.10
0.05
0
Δ ROC-AUC
H
Anisotropic - AP
AP - contact
DV - contact
mean fluorescence (A.U.)
200
150
100
50
0
10
20
30
40
time (min)

Figure S2. Contact length predicts transcription within neighbour-clusters. Related to Figure 3.

(A) Quantification of standard deviation of normalised apical areas for presumptive NBs (blue) versus non-NB neighbours (NCs, green) over the 2.5-minute window before transcription onset. Each cell was normalised using its initial apical area value within that time-window. SD is used a proxy for the amplitude of oscillations in apical area of each cell. N=30 clusters; 30 NBs, 44 NCs). Significance was assessed using a two-sample t-test, p-value shown on figure.

(B) Mean apical area of presumptive NBs (blue) and neighbours (NCs, green) plotted over a 2.5-minute window before transcription onset (t = 0). Area fluctuations of NBs and NCs are comparable across this time. Shaded regions denote ±SD. N=30 neuroblasts, 144 NCs).

(C) Representative time-lapse images of two smaller cells (blue) initially in contact during pre-transcription stages that then undergo dynamic contact-rearrangements over time to participate in adjacent clusters and delaminate separately.

(D) Receiver operating characteristic (ROC) analysis performed independently for NB–interface contact-length, contact-duration, and NC apical area to quantify their ability to discriminate transcribing from non-transcribing cells. The area under each ROC curve (AUC) was used as a threshold-independent measure of performance, NB-interface length (dark blue curve) emerged as strongest individual classifier (AUC= 0.76), contact-duration (light-blue curve) showed weak predictive ability (AUC = 0.55). NC apical area (grey-curve) appeared non-predictive (AUC= 0.52).

(E) Joint predictive performance was quantified using two supervised classification approaches, Logistic Regression and Random Forests. Models were trained using repeated stratified 70/30 train–test splits (N= 20). Performance was assessed using ROC-AUC values calculated on held-out test sets. 0.749 ± 0.067 for Logistic Regression, 0.711 ± 0.078 for Random Forest.

(F) Logistic regression PCA decision boundary shown for a representative test set. Green and blue regions denote areas predicted by the model as non-transcribing and transcribing, respectively. Test set data are overlaid (green squares, non-transcribing cells; blue squares, transcribing cells) and the majority are correctly classified.

(G) Permutation feature importance computed on held-out test sets for logistic regression and random forest models. For each feature, values were randomly permuted and the resulting decrease in model performance (ROC-AUC) was measured. Larger decreases indicate greater predictive contribution. Results identify NB–interface contact length as the dominant driver of model performance. Logistic Regression: Contact length 0.243 ± 0.052, Contact duration: 0.032 ± 0.029, Apical area -0.016 ± 0.025. Random Forest: Contact length 0.143 ± 0.048, contact duration: 0.044 ± 0.035, apical area -0.014 ± 0.027.

(H) Mean *E(spl)m8-MS2/MCP* fluorescence over time in anisotropic clusters with delaminating NB aligned to AP axis. Antero-posterior (AP; Grey) display higher transcriptional activity versus dorso-ventral aligned cells (DV; purple). Shaded region indicates SD.

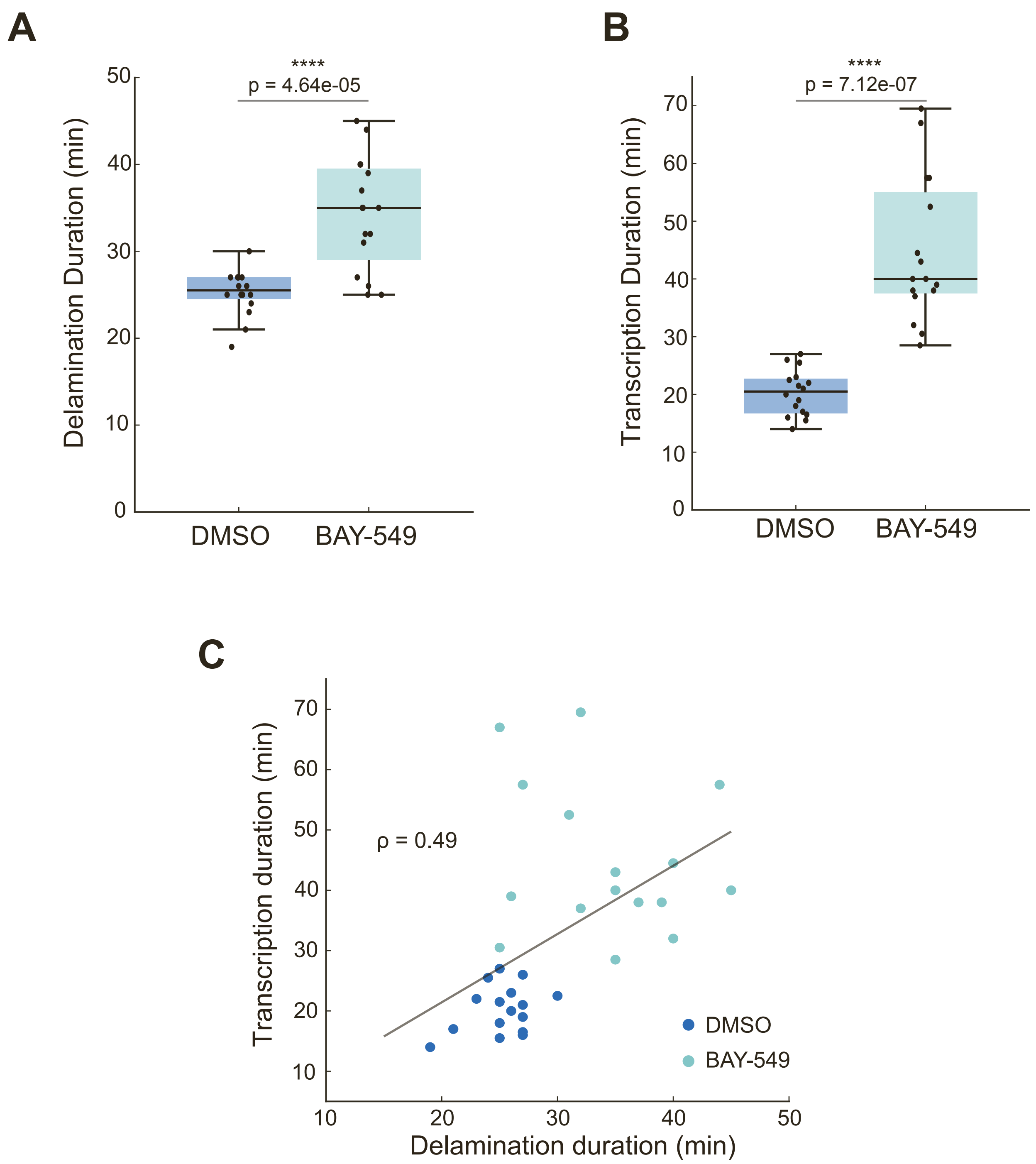
A
Delamination Duration (min)
****
p = 4.64e-05
0
10
20
30
40
50
DMSO
BAY-549
B
Transcription Duration (min)
****
p = 7.12e-07
0
10
20
30
40
50
60
70
DMSO
BAY-549
C
Transcription duration (min)
ρ = 0.49
10
20
30
40
50
60
70
DMSO
BAY-549
10
20
30
40
50
Delamination duration (min)

Figure S3. Delaying NB delamination via perturbing Myosin II based apical contractions prolongs transcription. Related to Figure 4.

(A) Time taken for NB to delaminate in DMSO (dark blue) and BAY549 ROCK-inhibitor (green) injected embryos, BAY-549 treatment significantly prolongs delamination duration N= 16 clusters for both DMSO and BAY549. Mean delamination durations: 25.25 ± 2.62, 34.25 ± 6.43 mins for DMSO and BAY549 respectively. p=4.64e-05 (two-sample t-test). n=16 clusters for both DMSO and BAY549.

(B) Total duration of *E(spl)m8–MS2* transcription within proneural clusters in DMSO (grey) and BAY-549 (blue) treated embryos Cluster transcriptional duration is prolonged in BAY-549 treated embryos compared to the controls. Mean transcription durations: 20.28 ± 3.96, 44.66 ± 12.5 mins for DMSO and BAY549 respectively. p=7.12e-07 (two-sample t-test). n=16 clusters for both DMSO and BAY549.

(C) Correlation between NB delamination and cluster transcription duration (as quantified in A and B) across DMSO and BAY549. Pearson correlation coefficient of 0.49 (p=0.0045) indicates positive correlation.

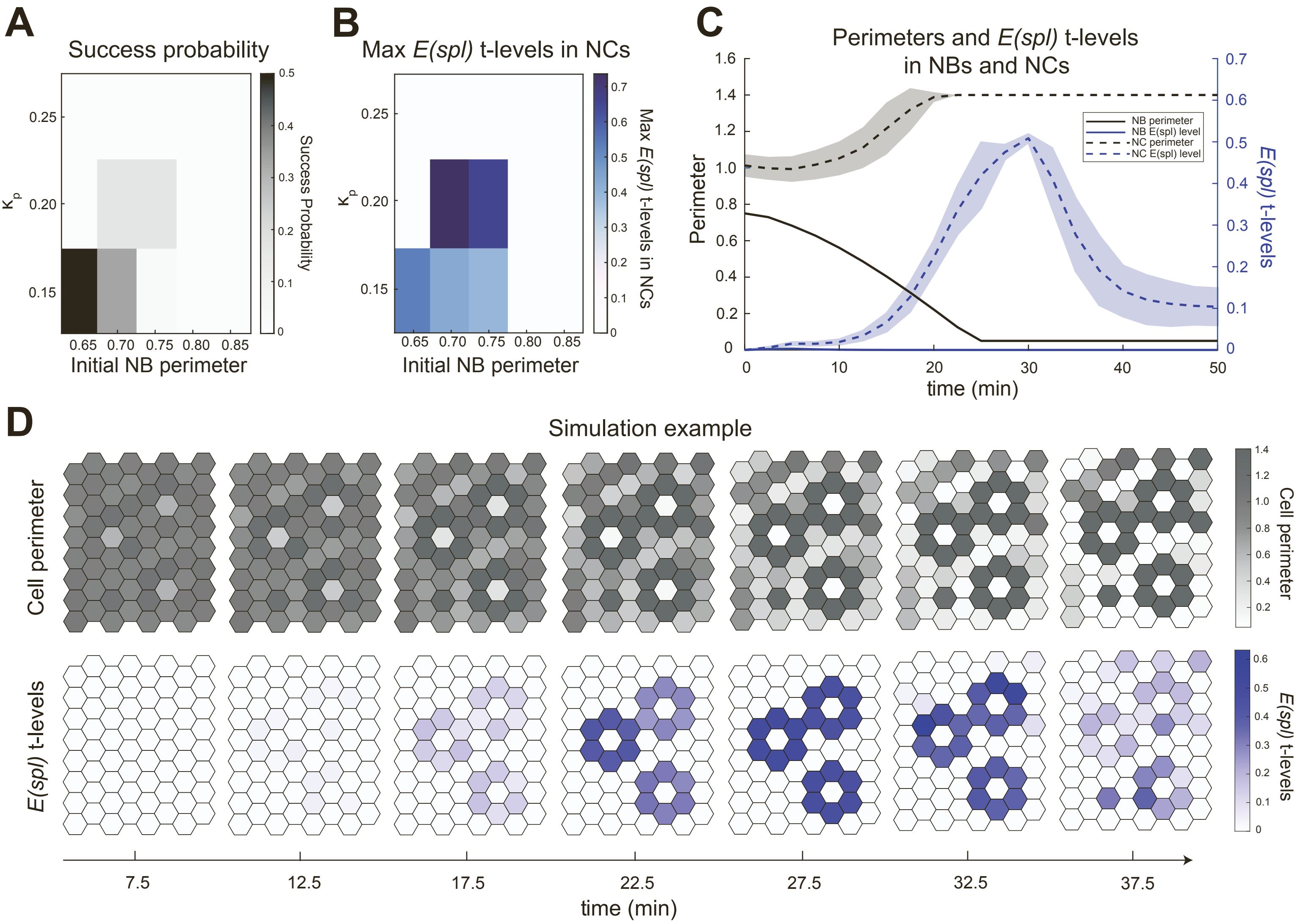
A
Success probability
Initial NB perimeter
Success Probability
B
Max E(spl) t-levels in NCs
Initial NB perimeter
C
Perimeters and E(spl) t-levels in NBs and NCs
Perimeter
E(spl) t-levels
time (min)
NB perimeter
NB E(spl) level
NC perimeter
NC E(spl) level
D
Simulation example
Cell perimeter
E(spl) t-levels
time (min)

Figure S4. Dynamic perimeter model reproduces many features of E(spl) transcription. Related to Figure 6.

(A) Heatmap of simulation success probabilities using a dynamic perimeter model, in which cell perimeter depends on *E(spl)* and activated Delta levels within each cell. Probabilities are averaged over different *E(spl)* detection threshold values (see Supplementary Information) and are calculated across values of the initial NB perimeter and κ_p, the parameter that scales the effect of *E(spl)* and activated Delta on perimeter in each cell (simulations duration= 25mins). Darker colours indicate higher success probability (see main text and Supplementary for success criteria).

(B) Heatmap of maximum *E(spl)* levels in NCs from simulations with dynamic perimeter model, in which cell perimeters depend on *E(spl)* and activated Delta levels, averaged over different *E(spl)* detection threshold values (see Supplementary Information). Maximum *E(spl)* levels are computed across values of the initial NB perimeter and κ_p. Darker colours indicate higher peak *E(spl)* expression (see main text and Supplementary Information for success criteria).

(C) Quantification of perimeter (grey) and *E(spl)* levels (blue) over time for NBs (solid curves) and NCs (dashed curves) from representative simulation with the dynamic perimeter model. As observed experimentally, NBs delaminate over ~25 min, and NC perimeters increase with *E(spl)* expression. Once delamination completes, *E(spl)* levels in NCs decline, consistent with experimental measurements. Only very low (below detection limit) *E(spl)* expression is observed in NBs. Parameters used for the simulation: *E(spl)* detection limit= 0.025, initial perimeter for NBs=0.75, initial perimeter for all other cells= 1 (± 5% noise, see Supplementary methos), γ_{cis}=0, γ_{tens}=3. Perimeters are restricted between [0.05,1.4], if a cell perimeter reaches the boundary, dp/dt for that cell is set to 0.

(D) Successful simulation of an 8×8 2D lattice corresponding to (C), showing temporal changes in cell perimeters (top panel, grey scale) and *E(spl)* levels (bottom panel, blue scale). Delamination of 3 NBs is accompanied by asynchronous *E(spl)* expression in NCs at varying amplitudes. Experimental observations are recapitulated up to ~27.5 min, when delamination concludes, beyond which the simulation diverges. In vivo NCs would enter into mitosis, disrupting cell contacts and trans-signalling.

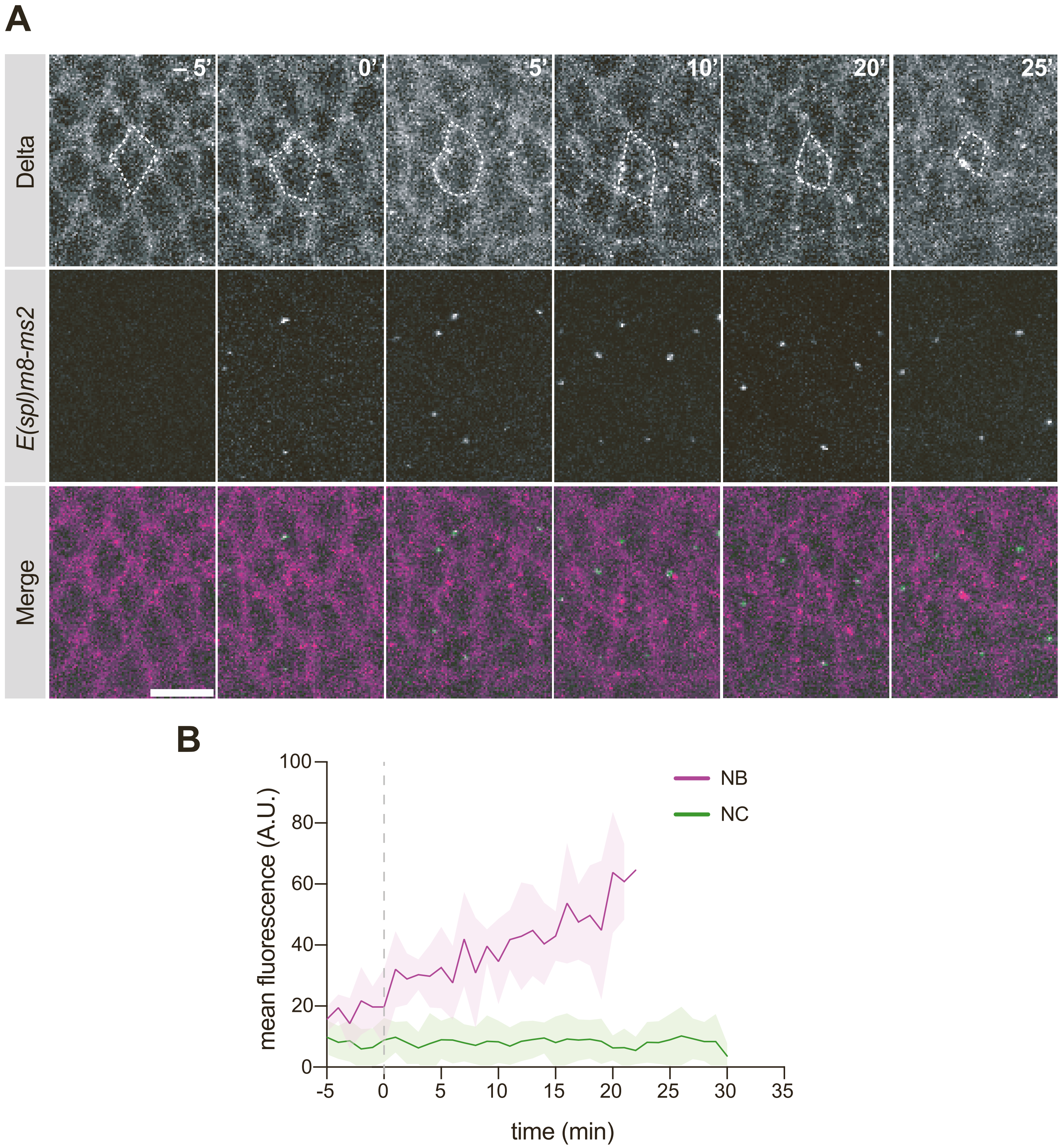
A
– 5’
0’
5’
10’
20’
25’
Delta
E(spl)m8-ms2
Merge
B
mean fluorescence (A.U.)
time (min)
NB
NC
100
80
60
40
20
0
-5
0
5
10
15
20
25
30
35

Figure S5. Changes in Delta enrichment during transcriptional progression. Related to Figure 6.

(A) Representative time-lapse images of Delta::mScarlet (top row) and *E(spl)m8–MS2* transcription (middle row) in a neighbour-cluster. Gradual enrichment of cytoplasmic Delta::mScarlet puncta occurs in NBs after transcription proceeds in NCs (Scale bar, 10 µm)

(B) Quantification of mean Delta fluorescence intensity over time in the NB (magenta) and neighbouring NCs (green), aligned to transcription onset (dashed line at 0'). Delta levels progressively increase in the NB post-onset of *E(spl)m8–MS2* transcription within the clusters, whereas NCs maintain relatively stable levels. Shaded regions indicate SD. (N=11 clusters).

Figure	Experiment	N	p Value	Statistical test used
Figure 1D	Individual *E(spl)m8-MS2* profile; Asynchronous transcription onset	N=1 cluster, 5 NCs	–	–
Figure 1E	Mean *E(spl)m8-MS2* fluorescence in clusters; NCs vs NBs	N=30 clusters, 30 NBs and 84 NCs	–	–
Figure 1F	NB apical area during cluster signalling	N=30 clusters, 30 NBs	–	–
Figure 1G	Mean *E(spl)m7-MS2* fluorescence in clusters; NCs vs NB	N=11 clusters, 11 NBs and 34 NCs	–	–
Figure S1B	*E(spl)m8-MS2* onset heatmap;	N=30 clusters, 84 NCs	–	–
Figure S1C	Transcription durations: *E(spl)m8-MS2* vs *E(spl)m7-MS2*	m8: N= 84 NCs;<br>m7: N= 58 NCs	3.30e-03	Two sample t-test
Figure S1E	*neur* mean fluorescence at the active transcription site.	N= 48 NB, 42 NC cells	0.1703	Two sample t-test
Figure 2B	*E(spl)m8-MS2* duration after NB ablation vs NC ablation	NB ablation: N=16 NCs;<br>NC ablation: N=26 NCs	–	–
Figure 2D	E(spl)m8-MS2 duration after contact ablation	NB-NC contact: N=10;<br>NC-NC contact: N=10	–	–
Figure 2G	Notch::Halo, Delta::mScarlet membrane fluorescence, NB or NC contacts	NB-NC contact: N=51 Notch::Halo; 53 Dl::mScarlet<br>NC-NC contact: N=67 Notch::Halo; 60 Dl::mScarlet	0.1288 for Notch; 0.0580 for Delta	Two sample t-test
Figure S2A	SD of normalised apical areas	N=30 clusters; 30 NBs, 144 NCs	p = 6.82e-02	Two sample t-test
Figure S2B	Mean apical areas of NB and NC pre onset of transcription	N=30 clusters; 30 NBs, 144 NCs	–	–
Figure S2D	ROC analysis: NB-interface contact length (AUC=0.76)	N=66 transcribing, 39 non-transcribing	AUC=0.76 (contact length), 0.55 (duration), 0.52 (area)	–
Figure S2F	Logistic Regression ROC-AUC	N=20	–	–
Figure S2H	Anisotropic AP delamination	N=8 clusters.<br>18 AP; 18 DV contact NCs	–	–
Figure 3C	Mean apical area: NB vs NC (2.5 min pre-transcription onset)	N=30 clusters; 30 NBs, 144 NCs	1.30e-03	Two sample t-test

Figure	Experiment	N	p Value	Statistical test used
Figure 3D	NB-NC contact length/apical area: transcribing vs non-transcribing NCs	N=66 transcribing, 39 non-transcribing NCs	2.06e-04	Two sample t-test
Figure 3E	NB-NC contact duration: transcribing vs non-transcribing NCs	N=66 transcribing, 39 non-transcribing NCs	9.19e-02 (n.s.)	Two sample t-test
Figure 3G	*E(spl)m8-MS2* fluorescence in NC with AP vs DV NB-contact in isotropic	N=22 isotropic clusters. 47 AP; 37 DV contact NCs	–	–
Figure 3H	*E(spl)m8-MS2* fluorescence in NC with AP vs DV NB-contact in anisotropic (DV) clusters	N=8 anisotropic clusters. 20 DV, 18 AP contact NCs	–	–
Figure 3I	*E(spl)m8-MS2* in NC with continuous vs transient NB contact in isotropic clusters	N=69 continuous, 40 transient NCs	–	–
Figure 3J	*E(spl)m8-MS2* in NC with continuous vs transient NB contact in anisotropic clusters	N=15 continuous, 20 transient NCs	–	–
Figure 4B	Displacement of tri-cellular junctions over time following contact ablation	N=10 NB-NC; 10 NC-NC contacts	–	–
Figure 4C	Initial recoil at 15s: NB-NC vs NC-NC contacts	N=10 NB-NC; 10 NC-NC contacts	2.39e-05	Two sample t-test
Figure 4E	NB delamination duration in y^{RNAi} vs $cyst^{RNAi}$ and cno^{RNAi}	N=10 (y^{RNAi}), 9 ($cyst^{RNAi}$), 11 (cno^{RNAi}) NBs	3.21e-08 for y^{RNAi} vs $cyst^{RNAi}$; 1.03e-04 for y^{RNAi} vs cno^{RNAi}	Two sample t-test
Figure 4F	*E(spl)m8-MS2* transcription duration in y^{RNAi} vs $cyst^{RNAi}$ and cno^{RNAi} clusters	N=10 (y^{RNAi}), 9 ($cyst^{RNAi}$), 11 (cno^{RNAi}) clusters	4.22e-06 for y^{RNAi} vs $cyst^{RNAi}$: 7.12e-05 for y^{RNAi} vs cno^{RNAi}	Two sample t-test
Figure 4G	Correlation: NB delamination duration vs transcription duration (all genotypes)	N=10 (y^{RNAi}), 9 ($cyst^{RNAi}$), 11 (cno^{RNAi}) clusters	Pearson correlation coefficient ρ=0.82, p=3.17e-08	t-test
Figure S3A	NB delamination duration: DMSO vs BAY-549 (ROCK inhibitor)	N=16 NB (each)	4.64e-05	Two sample t-test
Figure S3B	*E(spl)m8-MS2* transcription duration: DMSO vs BAY-549	N=16 clusters (each)	7.12e-07	Two sample t-test
Figure S3C	Correlation: delamination vs transcription duration (DMSO + BAY-549)	N=16 clusters (each)	Pearson correlation coefficient ρ=0.49, p=4.5e-03	t-test

Figure	Experiment	N	p Value	Statistical test used
Figure 5A	Cross-correlation: NB apical area vs E(spl)m8-MS2 intensity in NCs	N=30 NBs and 84 transcribing NCs	–	–
Figure 5B	Cross-correlation: transcribing NC apical area vs *E(spl)m8-MS2* intensity	N=84 transcribing NCs	–	–
Figure 5D	Apical area fold-change: transcribing and non-transcribing NCs, window 0-5 min post-onset	N=84 transcribing, 60 non-transcribing	4.02e-08, transcribing; 2.88e-1, non-transcribing	One-sample t-test on log2 transformed fold changes
Figure 5D	Apical area fold-change: transcribing NCs, window 5-10 min post-onset	N=84 transcribing, 60 non-transcribing	6.18e-12 for transcribing; 1.89e-02 for non-transcribing	One-sample t-test on log2 transformed fold changes
Figure 5E	Correlation: NC apical area vs area under MS2 curve	N=84 NCs	–	–
Figure 6B	Simulation success probability across γ_{cis} and γ_{tens}	8×8 2D hexagonal lattice simulations	–	–
Figure 6C	Max *E(spl)* levels in NCs across γ_{cis} and γ_{tens}	8×8 2D hexagonal lattice simulations	–	–
Figure S4D	Dynamic perimeter model lattice: asynchronous *E(spl)* in NCs	–	–	–
Figure S5B	Delta::mScarlet intensity over time: NB vs NCs aligned to transcription onset	N=11 clusters; 11 NBs, 21 NCs	–	–

Table S1. Statistical analysis and sample size of data presented in main and supplementary figures. Related to Figures 1–6 and Figures S1–S5.

Parameter	Description	Value
β_d	Delta production rate	1.62
β_n	Notch production rate	1.52
β_E	*E(spl)* production rate	1.62
β_A	Activator production rate	1.62
α^+	Delta activation rate	0.6
α^-	Delta de-activation rate	0.4
K_t	Trans-activation rate	0.8
K_c	Cis-inhibition rate	0.4
T_E	Threshold level for *E(spl)* to inhibit activator expression	0.0051
T_s	Threshold level for trans-activation signal to promote *E(spl)* expression	0.8
c_s	Hill coefficient for promoting *E(spl)* expression by trans-activation signal	3
γ_{tens}	Exponent scaling "tension" factor κ_{tens}	3 for Figures 6D,F and Figure S4
γ_{cis}	Exponent scaling cis-inhibition factor κ_{cis}	1 for Figures 6D,F<br>0 for Figure S4
t_0	Simulation timepoint that perimeter rule (44) is activated	0
τ	Delamination duration associated with perimeter rule (44)	10 for Figures 6B-E,<br>[5, 10, 15] for Figure 6F
$\mathrm{L_D}$	*E(spl)* detection threshold	[0.010 0.013 0.016 0.019] for Figures 6B,C<br>0.015 for Figures 6D,F<br>[0.015 0.02 0.025] for Figures S4A,B<br>0.025 for Figure S4C,D
κ_p	Ratio of Activated Delta and *E(spl)* Hill function prefactors in dynamic perimeter ODE (47)	[0.15, 0.20, 0.25] for Figures S4A, B<br>0.2 for Figures S4C,D
θ_δ	Threshold level for Delta activity to inhibit perimeter	0.4

θ_E	Threshold level for *E(spl)* expression to promote perimeter	0.4
h	Hill coefficient for inhibiting perimeter by Delta activity	2
m	Hill coefficient for promoting perimeter by *E(spl)* expression	1
d_0	Initial levels of Delta	1 ± 5% noise
d_0^A	Initial levels of activated Delta	0
n_0	Initial levels of activated Notch	1 ± 5% noise
E_0	Initial levels of *E(spl)*	0
A_0	Initial levels of activator	0.6 ± 5% noise
p_0	Initial perimeter values	0.85 for presumptive NB, 1 ± 5% noise for all other cells for Figures 6B-E<br>0.75 for presumptive NB, 1 ± 5% noise for all other cells for Figures S4C,D

Table S2. Initial values and parameters for model. Related to Figure 6 and S4.
