# Supplementary material for: Notch mediated lateral inhibition is shaped by morphological differences to reinforce bias toward signal-sending or receiving roles
Source: Curr Biol. Author manuscript; Available in PMC 2026 Sep 12. (PMC7619482; doi:10.1016/j.cub.2026.07.041)
Supplement: Supplementary file 2 [file EMS217835-supplement-Supplemental_Methods_S1.pdf]

Methods S1: Mathematical Appendix, related to STAR Methods

To examine how cell geometry and mechanical properties influence signalling dynamics, we modified the lateral inhibition model described by [M1], (which builds on the framework introduced by [M2]). Unlike the original formulation, our implementation does not rely on a spatial activator gradient. Instead, we defined a perimeter-weighted cell–cell connectivity matrix, such that the interaction strength between neighbouring cells is proportional to the length of their shared boundary.

To account for possible mechanical influences on signalling behaviour, we introduced two additional parameters that vary inversely with cell perimeter. The first parameter, κ_{tens}, represents a tension-related scaling factor that regulates the amount of activated Delta available to engage in trans-activation of Notch receptors on adjacent cells. The second parameter, κ_{cis}, controls the strength of cis-inhibition between Notch and Delta within the same cell (Figure 6A).

These extensions allow cell size to directly influence signalling dynamics within the model framework. In the following section, we present the full derivation of the model based on the formulation of [M1], incorporating the modifications described above.

Notation

n_i ≡ Notch levels in cell i
d_i ≡ Delta levels in cell i
d_i^A ≡ Activated Delta in cell i
S_i ≡ [NICD, Mam, Su(H)] complex levels in cell i
E_i ≡ *E(spl)* levels in cell i
A_i ≡ Activator levels in cell i
$\langle d_j \rangle_{\mathrm{i}}$ ≡ sum of Delta from cells j, on the boundaries with cell i
$\langle n_j \rangle_{\mathrm{i}}$ ≡ sum of Notch from cells j, on the boundaries with cell i
$[n_i d_j]$ ≡ complex of Notch from cell i and Delta from neighbour cell j
p_i ≡ perimeter of cell i

The following Michaelis-Menten reaction scheme corresponds to the processes illustrated in Figure 6A:

Delta activation

$$d_i + A_i \xrightarrow{\alpha^+} d_i^A + A_i \tag{1}$$

$$d_i^A \xrightarrow{\alpha^-} d_i \tag{2}$$

with α^+ and α^- the Delta activation and de-activation rates respectively.

Trans-activation, signal and transcriptional feedback

$$n_i + d_j \underset{\kappa^-}{\overset{\kappa^+}{\rightleftharpoons}} [n_i d_j] \tag{3}$$

$$n_i + d_j^A \underset{\kappa^-}{\overset{\kappa^+}{\rightleftharpoons}} [n_i d_j^A] \tag{4}$$

Assuming that only activated Delta can produce signal:

$$[n_i d_j^A] \xrightarrow{\kappa_{trans}} s_i \rightarrow E_i \dashv A_i \tag{5}$$

with κ^+ and κ^- the association and dissociation between Notch, Delta and the Notch-Delta complex. κ_{trans} is the trans-activation rate.

Cis-inhibition

Notch and Delta within the same cell can bind however the resulting complex does not lead to signal. Here we assume that the complex can either degrade or dissociate back to free Notch and Delta.

$$n_i + d_i \underset{\kappa_{cis}^{-non}}{\overset{\kappa_{cis}^{+non}}{\rightleftharpoons}} [n_i d_i] \xrightarrow{\kappa_{cis\_deg}^{non}} \emptyset \qquad (6)$$

$$n_i + d_i^A \underset{\kappa_{cis}^{-A}}{\overset{\kappa_{cis}^{+A}}{\rightleftharpoons}} [n_i d_i^A] \xrightarrow{\kappa_{cis\_deg}^{A}} \emptyset \qquad (7)$$

where κ_{cis}^{+non} and κ_{cis}^{+A} are association rates, κ_{cis}^{-non} and κ_{cis}^{-A} are dissociation rates while $\kappa_{cis\_deg}^{non}$ and $\kappa_{cis\_deg}^{A}$ are the degradation rates of cis-inhibited Delta-Notch complexes from non-activated and activated Delta respectively.

The reactions outlined above can be expressed as the following system of differential equations, using a Hill-function formulation:

Differential equations

$$\frac{dd_i}{dt} = \beta_d + \alpha^- d_i^A - \alpha^+ A_i d_i - \gamma_d d_i - \left(\kappa^+ \langle n_j \rangle_{\mathrm{i}} d_i - \kappa^- \left[\langle n_j \rangle_{\mathrm{i}} d_i\right]\right) - (\kappa_{cis}^{+non} n_i d_i - \kappa_{cis}^{-non} [n_i d_i]) \qquad (8)$$

$$\frac{dd_i^A}{dt} = \alpha^+ A_i d_i - \alpha^- d_i^A - \gamma_d^A d_i^A - \left(\kappa^+ \langle n_j \rangle_{\mathrm{i}} d_i^A - \kappa^- \left[\langle n_j \rangle_{\mathrm{i}} d_i^A\right]\right) - (\kappa_{cis}^{+A} n_i d_i^A - \kappa_{cis}^{-A} [n_i d_i^A]) \qquad (9)$$

$$\frac{dn_i}{dt} = \beta_n - \gamma_n n_i - \left(\left(\kappa^+ n_i \langle d_j \rangle_{\mathrm{i}} - \kappa^- \left[n_i \langle d_j \rangle_{\mathrm{i}}\right]\right) + \left(\kappa^+ n_i \langle d_j^A \rangle_{\mathrm{i}} - \kappa^- \left[n_i \langle d_j^A \rangle_{\mathrm{i}}\right]\right)\right) - ((\kappa_{cis}^{+non} n_i d_i - \kappa_{cis}^{-non} [n_i d_i]) + (\kappa_{cis}^{+A} n_i d_i^A - \kappa_{cis}^{-A} [n_i d_i^A])) \qquad (10)$$

$$\frac{d\left[n_i \langle d_j \rangle_{\mathrm{i}}\right]}{dt} = \kappa^+ n_i \langle d_j \rangle_{\mathrm{i}} - \kappa^- \left[n_i \langle d_j \rangle_{\mathrm{i}}\right] \qquad (11)$$

$$\frac{d\left[n_i \langle d_j^A \rangle_{\mathrm{i}}\right]}{dt} = \kappa^+ n_i \langle d_j^A \rangle_{\mathrm{i}} - \kappa^- \left[n_i \langle d_j^A \rangle_{\mathrm{i}}\right] - \kappa_{trans}^A \left[n_i \langle d_j^A \rangle_{\mathrm{i}}\right] \qquad (12)$$

$$\frac{dS_i}{dt} = \kappa_{trans}^A \left[n_i \langle d_j^A \rangle_{\mathrm{i}}\right] - \gamma_s S_i \qquad (13)$$

$$\frac{dE_i}{dt} = \frac{\beta_E \left(\frac{S_i}{T_s}\right)^{c_s}}{1 + \left(\frac{S_i}{T_s}\right)^{c_s}} - \gamma_E E_i \qquad (14)$$

$$\frac{dA_i}{dt} = \frac{\beta_A}{1 + \left(\frac{E_i}{T_E}\right)^{c_E}} - \gamma_A A_i \qquad (15)$$

$$\frac{d[n_i d_i]}{dt} = \kappa_{cis}^{+non} n_i d_i - \kappa_{cis}^{-non} [n_i d_i] - \kappa_{cis\_deg}^{non} [n_i d_i] \qquad (16)$$

$$\frac{d[n_i d_i^A]}{dt} = \kappa_{cis}^{+A} n_i d_i^A - \kappa_{cis}^{-A} [n_i d_i^A] - \kappa_{cis\_deg}^{A} [n_i d_i^A] \qquad (17)$$

$\beta_d, \beta_n, \beta_E, \beta_A$ are expression rates of Delta, Notch, *E(spl)* and activator respectively,

$\gamma_d, \gamma_n, \gamma_s, \gamma_E, \gamma_A$ are degradation rates of Delta, Notch, trans-activation signal, *E(spl)* and activator respectively,

T_s and T_E are the Hill half occupations levels associated with trans-activation signal promoting *E(spl)* expression and *E(spl)* inhibiting activator expression respectively, while c_S and c_E are the respective Hill coefficients.

Assuming that association rates of cis-inhibitory complexes are the same for all Delta variations:

Let $\kappa_{cis}^{-non} = \kappa_{cis}^{-A} = \kappa_{cis}^{-}$, $\kappa_{cis}^{+non} = \kappa_{cis}^{+A} = \kappa_{cis}^{+}$ and $\kappa_{cis\_deg}^{non} = \kappa_{cis\_deg}^{A}$

Assuming steady-state for Delta-Notch & [NICD, Mam, Su(H)] complexes:

$$\frac{d[n_i\langle d_j\rangle_{\mathrm{i}}]}{dt} = 0 \Rightarrow [n_i\langle d_j\rangle_{\mathrm{i}}] = \frac{\kappa^+}{\kappa^-} n_i\langle d_j\rangle_{\mathrm{i}} \ ; \ [\langle n_j\rangle_{\mathrm{i}} d_i] = \frac{\kappa^+}{\kappa^-} \langle n_j\rangle_{\mathrm{i}} d_i \tag{18}$$

$$\frac{d[n_i\langle d_j^A\rangle_{\mathrm{i}}]}{dt} = 0 \Rightarrow [n_i\langle d_j^A\rangle_{\mathrm{i}}] = \frac{\kappa^+}{\kappa^- + \kappa_{trans}^A} n_i\langle d_j^A\rangle_{\mathrm{i}} \tag{19}$$

$$[\langle n_j\rangle_{\mathrm{i}} d_i^A] = \frac{\kappa^+}{\kappa^- + \kappa_{trans}^A} \langle n_j\rangle_{\mathrm{i}} d_i^A \tag{20}$$

$$\frac{d[n_i d_i]}{dt} = 0 \Rightarrow [n_i d_i] = \frac{\kappa_{cis}^+}{\kappa_{cis}^- + \kappa_{cis\_deg}} n_i d_i \tag{21}$$

$$\frac{d[n_i d_i^A]}{dt} = 0 \Rightarrow [n_i d_i^A] = \frac{\kappa_{cis}^+}{\kappa_{cis}^- + \kappa_{cis\_deg}} n_i d_i^A \tag{22}$$

$$\frac{ds_i}{dt} = 0 \Rightarrow S_i = \frac{\kappa_{trans}^A}{\gamma_s} [n_i\langle d_j^A\rangle_{\mathrm{i}}] \tag{23}$$

$$\Rightarrow S_i = \frac{\kappa_{trans}^A}{\gamma_s} \frac{\kappa^+}{\kappa^- + \kappa_{trans}^A} n_i\langle d_j^A\rangle_{\mathrm{i}} \tag{24}$$

Let

$$K_t \equiv \frac{\kappa^+\kappa_{trans}^A}{\kappa^- + \kappa_{trans}^A} \tag{25}$$

be trans-activation rate and

$$K_c \equiv \frac{\kappa_{cis}^+ \kappa_{cis\_deg}}{\kappa_{cis}^- + \kappa_{cis\_deg}} \tag{26}$$

be cis-inhibition rate.

New equations after the above assumptions and definitions:

$$\frac{dd_i}{dt} = \beta_d + \alpha^- d_i^A - \alpha^+ A_i d_i - (\gamma_d + K_c n_i) d_i \tag{27}$$

$$\frac{dd_i^A}{dt} = \alpha^+ A_i d_i - (\alpha^- + \gamma_d^A + K_t\langle n_j\rangle_{\mathrm{i}} + K_c n_i) d_i^A \tag{28}$$

$$\frac{dn_i}{dt} = \beta_n - \gamma_n n_i - K_t n_i\langle d_j^A\rangle_{\mathrm{i}} - K_c(d_i + d_i^A) n_i \tag{29}$$

$$\frac{dE_i}{dt} = \frac{\beta_E \left(\frac{K_t}{\gamma_s T_s}\right)^{c_s} \left(n_i\langle d_j^A\rangle_{\mathrm{i}}\right)^{c_s}}{1 + \left(\frac{K_t}{\gamma_s T_s}\right)^{c_s} \left(n_i\langle d_j^A\rangle_{\mathrm{i}}\right)^{c_s}} - \gamma_E E_i \tag{30}$$

$$\frac{dA_i}{dt} = \frac{\beta_N}{1 + \left(\frac{E_i}{T_E}\right)^{c_E}} - \gamma_A A_i \tag{31}$$

Non-dimensionalization

As in [M1], we normalize all of the levels by the initial Notch level, n_0, and scale the time by the *E(spl)* degradation rate, γ_E, assuming degradation rates are equal among variables:

$$\gamma_d = \gamma_n = \gamma_s = \gamma_E = \gamma_A$$

Dimensionless equations after simplifications are:

$$\frac{dd_i}{dt} = \beta_d + \alpha^- d_i^A - \alpha^+ A_i d_i - (1 + K_c n_i) d_i \tag{32}$$

$$\frac{dd_i^A}{dt} = \alpha^+ A_i d_i - (\alpha^- + 1 + K_t \langle n_j \rangle_\mathrm{i} + K_c n_i) d_i^A \tag{33}$$

$$\frac{dn_i}{dt} = \beta_n - n_i - K_t n_i \langle d_j^A \rangle_\mathrm{i} - K_c (d_i + d_i^A) n_i \tag{34}$$

$$\frac{dE_i}{dt} = \frac{\beta_E \left(\frac{K_t}{T_s} n_i \langle d_j^A \rangle_\mathrm{i}\right)^{c_s}}{1 + \left(\frac{K_t}{T_s} n_i \langle d_j^A \rangle_\mathrm{i}\right)^{c_s}} - E_i \tag{35}$$

$$\frac{dA_i}{dt} = \frac{\beta_A}{1 + \left(\frac{E_i}{T_E}\right)^{c_E}} - A_i \tag{36}$$

Introducing perimeters

A perimeter is introduced for each cell and the connectivity matrix (which determines the function of Notch and Delta on boundaries between cells) is weighed using these perimeters.
The original connectivity matrix (in [M1]):

$$C_{ij} = \begin{cases} \frac{1}{6}, & if\ i\ and\ j\ are\ neighbors \\ 0, & otherwise \end{cases} \tag{37}$$

is adapted using estimations of the contact lengths between cells using their perimeters:

$$L_{ij} = \frac{\frac{1}{2}(p_i + p_j)}{\langle \frac{1}{2}(p_i + p_j) \rangle_{\mathrm{C_{ij>0}}}} \tag{38}$$

$$C_{ij}^w = C_{ij} L_{ij} \tag{39}$$

Hypothesizing that cis-inhibition is inversely related to cell-size potentially reflecting membrane crowding effects that limit the availability of free Notch, we introduce perimeter dependent parameter κ_{cis}, as

$$\kappa_{\mathrm{cis,i}} = \left(\frac{\langle \mathrm{p} \rangle}{p_i}\right)^{\gamma_{cis}} \tag{40}$$

which scales the cis-inhibition factor as follows:

$$K_{c,i} = \kappa_{\mathrm{cis,i}} K_c \tag{41}$$

We also introduce a perimeter-based tension factor, κ_{tens}, based on the hypothesis that higher tension in smaller cells results in more activated Delta at their boundaries (for example via effects on endocytosis) available for trans-activation neighbouring Notch:

$$\kappa_{\mathrm{tens,i}} = \frac{\left(\frac{\langle \mathrm{p} \rangle}{\mathrm{p_i}}\right)^{\gamma_{tens}}}{\langle \left(\frac{\langle \mathrm{p} \rangle}{\mathrm{p_i}}\right)^{\gamma_{tens}} \rangle} \tag{42}$$

This factor re-scales the amount of activated Delta available for trans-activation as follows:

$$\langle d_j^A \rangle_{\mathrm{i}} = \sum_j C_{ij}^w \kappa_{\mathrm{tens,j}} d_j^A \tag{43}$$

For the non-dynamic, imposed perimeter model shown in Figure 6, we use the following perimeter rules:

(i) a smooth, time-dependent decrease in NB perimeter and

(ii) an increase in perimeter in cells whose *E(spl)* levels exceeded the *E(spl)* detection threshold as follows:

$$p_i(t) = \big(1 - s(t)\big) p_{0,i} + s(t) p_{target,i} \tag{44}$$

where,

$$s(t) = \begin{cases} 0 & t < t_0 \\ \dfrac{t - t_0}{\tau} & t_0 \le t < t_0 + \tau \\ 1 & t \ge t_0 + \tau \end{cases} \tag{45}$$

and

$$p_{target,i} = \begin{cases} 0.05 & for\ NB \\ 1.4 p_{0,i} & if\ E_i > \mathrm{L_D} \\ p_{0,i} & otherwise \end{cases} \tag{46}$$

where p_i is the perimeter of cell i (with $p_{0,i}$ the initial perimeter),
τ is the delamination duration and t_0 the timepoint when this rule is triggered
and $\mathrm{L_D}$ is *E(spl)* detection limit.

To test whether our model would work with fully dynamic perimeter behaviour rather than imposed changes (Figure S4), we introduce a perimeter non-dimensionalised ordinary differential equation. As before, time is scaled by the *E(spl)* degradation rate, γ_E. Perimeter evolution is represented as a function of intracellular *E(spl)* and activated Delta levels using a Hill function formulation:

$$\frac{dp_i}{dt} = -\kappa_p \frac{d_{A,i}^h}{\theta_\delta^h + d_{A,i}^h} + \frac{E_i^m}{\theta_E^m + E_i^m} \tag{47}$$

where κ_p is the ratio of Activated Delta and *E(spl)* Hill function prefactors, θ_δ and θ_E are the Hill activation thresholds associated with Delta activity inhibiting perimeter and *E(spl)* promoting perimeter respectively, while h and m and the respective Hill coefficients.

Computer simulation

The dimensionless equations are solved numerically, using a standard Matlab ODE solver, and the solution is simulated on a 8x8 2D hexagonal lattice (Figure 6D) using a periodic boundaries condition.

For the model with imposed perimeter changes (Figure 6A) the following success criteria are used:

(i) *E(spl)* expression occurred only in NCs, with at least one NC exceeding the detection threshold and (ii) the ratio between maximal *E(spl)* levels in NCs and the detection threshold exceeded 10, consistent with calibration of our MS2 system [M3].

For the model with dynamic perimeter (Figure S4) two additional conditions are added:

(i) NB perimeter had to decrease below 0.3 by the end of the simulation, and
(ii) the mean perimeter of non-NB, non-NC cells had to remain above 0.6.

Following our non-dimensionalization procedure, as described above, the time in the simulation is in units of the typical half-life of key proteins in the system (estimated to be ~2.5mins) [M4].
Parameters in Table S2 are used, with parameters from the original model retaining the values reported in that study (see [M1] for justification).

Mathematical appendix references

M1. Troost, T., Binshtok, U., Sprinzak, D., and Klein, T. (2023). Cis-inhibition suppresses basal Notch signaling during sensory organ precursor selection. Proc. Natl. Acad. Sci. U. S. A. *120*, e2214535120. https://doi.org/10.1073/pnas.2214535120.

M2. Sprinzak, D., Lakhanpal, A., Lebon, L., Santat, L.A., Fontes, M.E., Anderson, G.A., Garcia-Ojalvo, J., and Elowitz, M.B. (2010). Cis-interactions between Notch and Delta generate mutually exclusive signalling states. Nature 2010 465:7294 *465*, 86–90. https://doi.org/10.1038/nature08959.

M3. Santa-Cruz Mateos, C., Roussos, C., de Haro Arbona, F.J., Falo-Sanjuan, J., and Bray, S. (2026). Direct observation of Notch signaling–induced transcription hubs mediating gene-expression responses. Sci. Adv. *12*. https://doi.org/10.1126/sciadv.aea5664.

M4. Housden, B.E., Fu, A.Q., Krejci, A., Bernard, F., Fischer, B., Tavaré, S., Russell, S., and Bray, S.J. (2013). Transcriptional Dynamics Elicited by a Short Pulse of Notch Activation Involves Feed-Forward Regulation by E(spl)/Hes Genes. PLoS Genet. *9*, e1003162. https://doi.org/10.1371/JOURNAL.PGEN.1003162.
