# Supplementary material for: Notch mediated lateral inhibition is shaped by morphological differences to reinforce bias toward signal-sending or receiving roles
Source: Curr Biol. Author manuscript; Available in PMC 2026 Sep 12. (PMC7619482; doi:10.1016/j.cub.2026.07.041)
Supplement: Supplementary file 3 [file EMS217835-supplement-Supplementary_videos.pdf]

Document S1. Figures S1-S5, Tables S1, S2.

Methods S1: Mathematical Appendix, related to STAR Methods

Supplementary videos:

Video S1: *E(spl)m8–MS2* and *E(spl)m7–MS2* transcription dynamics in ventral ectoderm. Related to Figure 1, Figure S1C.

Video S2: Transcriptional activity within a cluster in relation to NB delamination. Related to Figure 1, Figure S1.

Video S3: Effects of ablations on transcription activity. Related to Figure 2D F.

Video S4: Effects of RNAi depletions. Related to Figure 4D–G.

Video S5: Results from modelling simulation. Related to Figure 6.
